# Expression profile analysis of early fruit development in *iaaM*-parthenocarpic tomato plants

**DOI:** 10.1186/1756-0500-2-143

**Published:** 2009-07-21

**Authors:** Barbara Molesini, Giuseppe L Rotino, Angelo Spena, Tiziana Pandolfini

**Affiliations:** 1 Dipartimento di Biotecnologie, University of Verona, Strada Le Grazie 15, 37134-Verona, Italy; 2 CRA-ORL Agricultural Research Council Research Unit for Vegetable Crops, Via Paullese 28, 26836-Montanaso Lombardo, Italy; 3 Dipartimento di Scienze, Tecnologie e Mercati della Vite e del Vino, University of Verona, Via della Pieve 70, 37029-San Floriano (Verona), Italy

## Abstract

**Background:**

Fruit normally develops from the ovary after pollination and fertilization. However, the ovary can also generate seedless fruit without fertilization by parthenocarpy. Parthenocarpic fruit development has been obtained in tomato (*Solanum lycopersicum*) by genetic modification using auxin-synthesising gene(s) (*DefH9-iaaM*; *DefH9-RI-iaaM*) expressed specifically in the placenta and ovules.

**Findings:**

We have performed a cDNA Amplified Fragment Length Polymorphism (cDNA-AFLP) analysis on pre-anthesis tomato flower buds (0.5 cm long) collected from *DefH9-iaaM *and *DefH9-RI-iaaM *parthenocarpic and wild-type plants, with the aim to identify genes involved in very early phases of tomato fruit development. We detected 212 transcripts differentially expressed in auxin-ipersynthesising pre-anthesis flower buds, 65 of them (31%) have unknown function. Several differentially expressed genes show homology to genes involved in protein trafficking and protein degradation via proteasome. These processes are crucial for auxin cellular transport and signaling, respectively.

**Conclusion:**

The data presented might contribute to elucidate the molecular basis of the fruiting process and to develop new methods to confer parthenocarpy to species of agronomic interest. In a recently published work, we have demonstrated that one of the genes identified in this screening, corresponding to #109 cDNA clone, regulates auxin-dependent fruit initiation and its suppression causes parthenocarpic fruit development in tomato.

## Introduction

Tomato (*Solanum lycopersicum*) fruit represents an important component of the human diet due to high content in fibres, vitamins and antioxidants. The identification of genes that control growth and maturation of tomato fruit will allow their manipulation, by breeding and/or genetic engineering, to improve fruit quality. Biochemical and genetic aspects of late stages of fruit development, in particular ripening, have been widely investigated [1-4], while initial phases of fruit development have received less attention, despite their importance for both basic and applied research. Until now, few studies have been performed to investigate tomato fruit set and early development (1 to 15 days post anthesis) [5-7].

In Angiosperms, once a flower is pollinated and fertilization successfully takes place, ovary starts to grow and this is the first visible sign of fruit development [8]. The earliest phase of fruit growth is referred to as fruit set or fruit initiation.

In parthenocarpic plants, fruit set and development occurs without fertilization leading to the production of seedless fruits. The first phytohormone shown to trigger parthenocarpic fruit development was auxin applied exogenously to tomato flowers [9]. It is extensively demonstrated that one of the possible methods for achieving parthenocarpic fruit development, employing genetic engineering, is based on the ovary-specific expression (driven by *DefH9 *promoter) of *iaaM *and *RI-iaaM *genes, which code for an enzyme of the auxin biosynthetic pathway [10-13]. The two chimeric genes, *DefH9-iaaM *and *DefH9-RI-iaaM*, differ *in vitro *in their translational potential, and *in planta *in the level of auxin (total IAA) content in flower buds [12]. *RI-iaaM *is an *iaaM *derivative, modified in its 5'ULR in order to down-regulate the level of expression. Both *DefH9-iaaM *and *DefH9-RI-iaaM *parthenocarpic tomato flower buds are characterised by an increased content of auxin in female gametophyte as compared with wild-type [10-12], but *DefH9-iaaM*-expressing flower buds contain 5 times higher IAA than *DefH9-RI-iaaM*-expressing flower buds [12]. At pre-anthesis the *iaaM*-parthenocarpic flower buds appear morphologically identical to those of wild-type, except for an early enlargement of the ovary [14], indicating that high levels of auxin can derepress ovary growth before fertilization. Therefore, *iaaM*-parthenocarpic plants show precocious fruit growth. It is reasonable to assume that in parthenocarpic flower buds, the genetic program for fruit development has been already switched on during early stages of flower development, well before anthesis. On this premises, the *iaaM*-parthenocarpic flower buds represent a suitable experimental model to study molecular events taking place during the early phases of fruit growth, monitoring expression changes that occur mainly within the ovary.

## Results and discussion

A cDNA-AFLP approach [15] was used to generate expression profiles of young flower buds isolated from four independent parthenocarpic UC82 tomato lines, two transgenic for *DefH9-iaaM *(#3 and #2; see [12] for a description of the lines) and two for *DefH9-RI-iaaM *gene (#s5 and #s6; see [12] for a description of the lines), and from wild-type plants. Flower buds under analysis (0.5 cm in length) were at a very early stage of development (6–7 days before anthesis).

**Figure 1:**
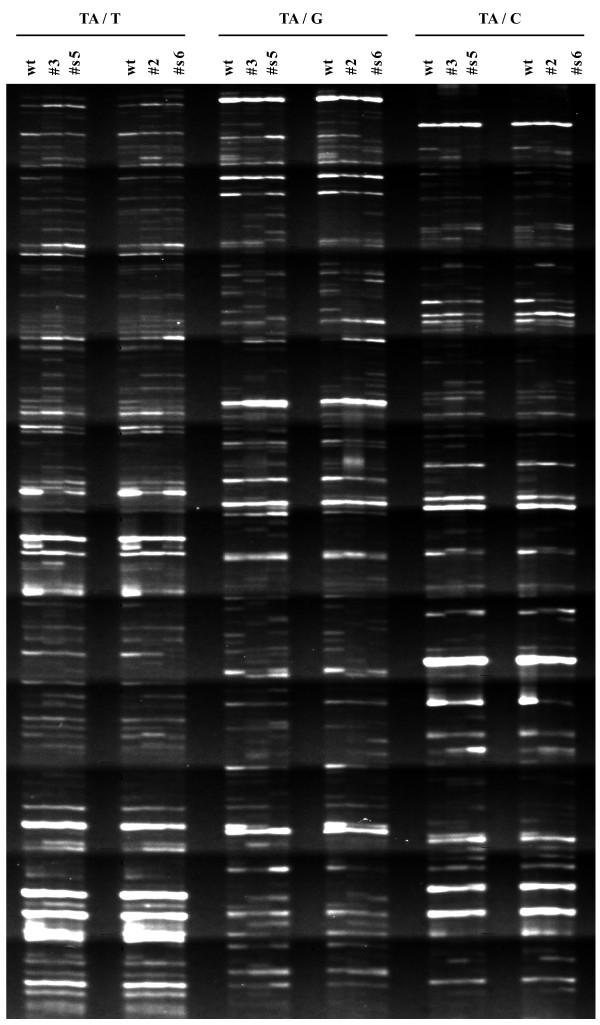
**Portion of a typical cDNA-AFLP gel**. An example of gel showing selective amplification with 3 different primer combinations (BstTA/MseT; BstTA/MseG and BstTA/MseC). Expression profiles of flower buds (0.5 cm long) from wild-type plants, two independent *DefH9-iaaM *lines (#3, # 2), and two independent *DefH9-RI-iaaM *lines (#s5, #s6) are reported. PCR products were size-fractionated on a 6% urea-polyacrylamide gel.

Using 32 different primer combinations (BstT/C+n – Mse+n, where n represents selective nucleotide) more than 3000 cDNA fragments were generated. The AFLP fragments ranged in length from 50 to 500 bp; for each primer combination, approximately 100 bands were observed on polyacrylamide denaturing gel (Figure 1). The differentially expressed fragments were excised from the gels, re-amplified by PCR and sequenced. Good quality and unique sequences were obtained for 212 cDNA fragments (Additional files 1 and 2).

The expression profiles of the isolated fragments were reproducible in the independent transgenic lines chosen for the analysis (Figure 1). One hundred and thirty cDNAs out of the 212 differentially expressed were induced in *iaaM*-parthenocarpic lines as compared to wild-type, whilst the remaining 82 cDNAs were down-regulated.

**Figure 2:**
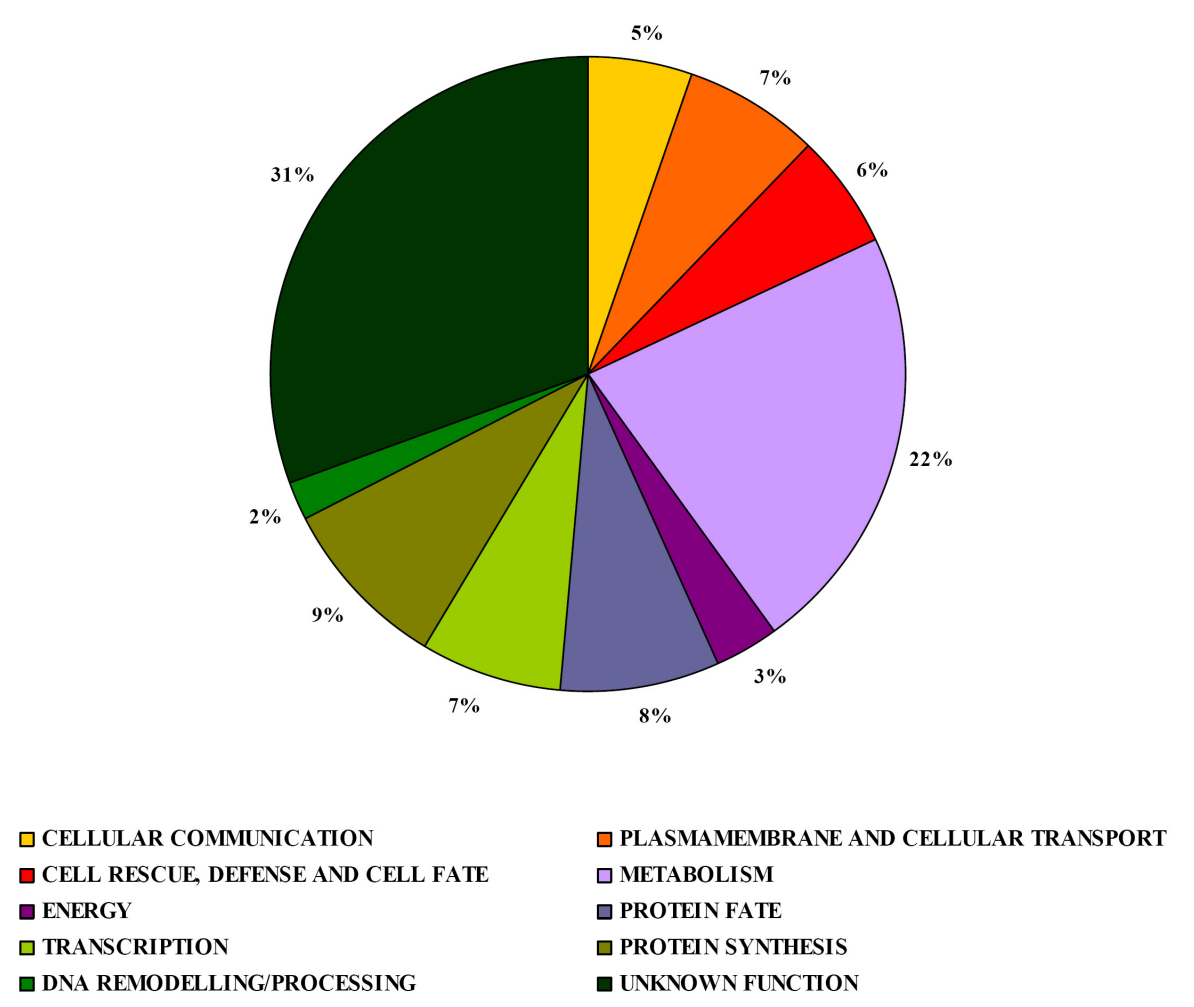
**Functional distribution of differentially expressed genes**. Genes were classified into 10 functional categories based on their putative function.

We performed quantitative RT-PCR analysis to validate some of the differentially expressed gene (Additional file 3) representative of the different functional categories (Additional file 2), using RNA extracted from independent parthenocarpic lines different from those tested by cDNA-AFLP. The sequences of the 212 differentially expressed genes were compared with those in GenBank database , DNA Data Bank of Japan database  and DFCI Tomato Unique Gene Indices , using BlastN or BlastX homology search tool [16]. The differentially expressed cDNA-AFLP clones are listed in additional file 2; for each clone the accession number, if available, and the e-value of the sequence that produced the highest identity score, together with the best *S. lycopersicum *Tentative Consensus (TC) sequence (DFCI Tomato Unique Gene Indices), are reported. A tentative annotation was assigned to about 70% of the cDNA-AFLP clones following GenBank and DFCI Tomato Unique Gene Indices databases. The cDNA clones were grouped in 10 functional categories (Figure 2) according to Universal Protein Resource (UniProt)  and TAIR database .

The two most represented categories "metabolism" and "unknown function" contain 47 genes (22%) and 65 genes (31%), respectively (Figure 2 and additional file 2). The majority of genes of the first category include enzymes with primary metabolic roles (e.g. phospholipid, carbohydrate and amino-acid metabolism). Other genes belonging to this category code for enzymes involved in secondary metabolism, such as spermidine synthase (#376) and ornithine decarboxylase (#216) that are up-regulated in *iaaM*-parthenocarpic flower buds and participate in the polyamine biosynthetic pathway. This finding is consistent with previous data showing that polyamines play a role in early fruit development [17] and it is in accordance with the experimental evidence demonstrating that polyamine metabolism is affected by exogenous auxin treatment of tomato ovaries [18].

In the functional category "Cell rescue, Defense and Cell fate" most of the genes involved in biotic stress showed a decreased expression in parthenocarpic flower buds. This is in accordance with the observation that tomato unpollinated flowers are actively protected against pathogens whereas after induction of fruit growth the expression of defence genes is reduced [7].

*iaaM*-parthenocarpic tomato flower buds have higher auxin levels compared with wild-type flower buds [12]. The increased indole-3-acetic acid (IAA) content in ovaries and placenta is considered to mimic the burst of auxin, which occurs in the carpel after pollination [19]. Interestingly, one of the genes in the category "metabolism", cDNA clone #436 induced in *iaaM*-parthenocarpic flower buds, codes for a molybdenum cofactor sulfurase protein-like, an enzyme taking part in the molybdenum cofactor biosynthetic pathway [20]. The molybdenum cofactor sulfurase ABA3 from *Arabidopsis thaliana *is necessary for post-translational activation of aldehyde oxidase (AOs) and xanthine dehydrogenase (XDH) [21]. In plants, AOs form a multigene family whose members have broad substrate specificity for several aldehydes including indole-3-acetaldehyde and abscisic aldehyde suggesting for AOs a role in the biosynthesis of IAA and abscisic acid (ABA). Other experimental evidences linked AO activity to the biosynthesis of IAA. AO1 activity in IAA-overproducing *Arabidopsis *plants is 5 times higher as compared to wild-type plants [22]. The up-regulation of #436 might contribute to increase IAA content observed in *iaaM*-parthenocarpic flower buds. However, we cannot exclude that the modulation of this gene might be related to ABA biosynthetic activity. In this regard, genes involved in ABA biosynthesis were shown to be strongly expressed in tomato ovary collected from flowers at anthesis [7].

The molecular events underlying the auxin-mediated derepression of ovary growth have not been fully elucidated. The augmented synthesis of auxin could affect IAA signaling pathway and/or modify the expression and localization of auxin transporters [23].

Auxin is perceived by intracellular located TIR1/AFB1–3 receptors that mediate the ubiquitination of AUX/IAA proteins that are then degraded by 26S-proteasome [23-25]. Most of the differentially expressed genes present in the "protein fate" functional category show homology to genes involved in proteolytic degradation via ubiquitination.

The establishment of auxin gradients in tissue/organs is crucial for auxin action [26-28] and requires the polar distribution of transporters in the plasma membrane. There is experimental evidence proving that the flexible asymmetric localization of IAA transporters is assured by cycling of these proteins between plasma membranes and endosome compartments [26-28], however many aspects of this regulatory process are still elusive.

In this regard, in the category "Plasmamembrane and cellular transport" a number of differentially expressed transcripts are homologous to genes implicated in intracellular protein transport. cDNA-AFLP clone #163 is similar to Rab8 of *Nicotiana tabacum*, putatively involved in protein movement between Golgi apparatus and the plasma membrane; #438 is homologous to SEC61 a protein translocator of endoplasmic reticulum (RE); #877 is similar to synthaxin-71, a component of the SNARE complex which functions in vesicles trafficking; #408 is annotated as μ-subunit of the clathrin adaptor complex. Furthermore, #303 clone encodes for a protein with not yet identified function, characterised by the clathrin-box motif (L [IVLMF]X [IVLMF] [DE]) (Eukaryotic Linear Motif (ELM) ). It would be interesting to investigate whether the differentially expressed genes found to be implicated in intracellular protein trafficking, play a role in the distribution of auxin transporters during early stage of fruit development. In fact, the treatment of tomato flower buds with inhibitors of polar auxin transport induces parthenocarpic fruit development [8].

**Figure 3:**
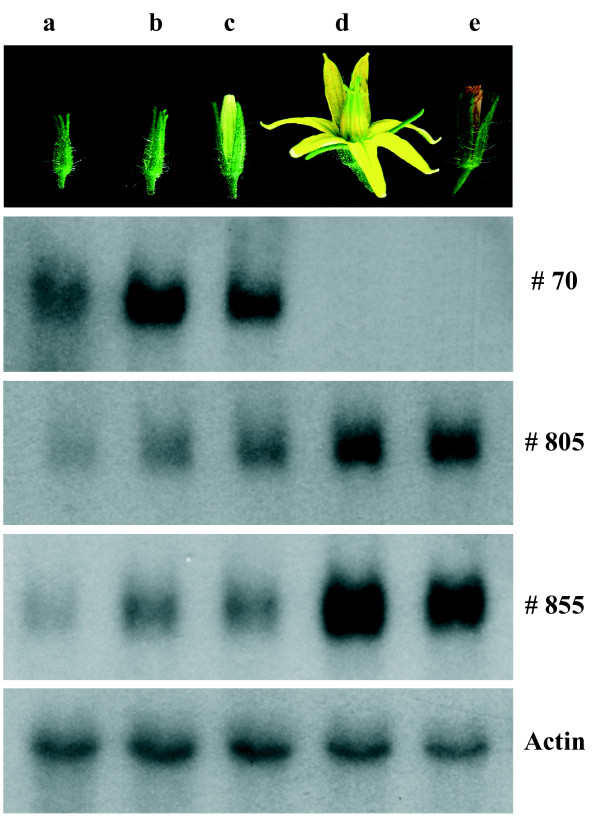
**Expression pattern of differentially expressed transcripts at different stages of wild-type flower development**. (Upper panel) a, b, c: flower buds at 6–7, 4–5 and 1–3 days before anthesis, respectively; d: open flower (approximately 2 days after anthesis); e: flower 4–5 days after anthesis. (Bottom panels) Northern blot analysis of mRNAs corresponding to #70, #805 and #855 cDNA-AFLP clones. The filter was also hybridised to an actin probe.

Genes involved in fruit set and/or early fruit development are often transcriptionally regulated during the first phases of ovary growth. We have investigated in wild-type tomato plants the expression of some genes identified in this screening (cDNA clones #70, #855, #805 reported in Figure 3; #109, see [14]; #216 and #904, see [13]) at different stages of flower and fruit development. These genes show a drastic increase (#855, and #805) or decrease (#70, #109, #216, and #904) in transcript level at the stage of open flower. Considering their expression profiles, it would be reasonable to test these genes for their putative role in fruit development. For instance, cDNA clone #109 (named by us *SlAucsia*-1 gene; [14]) and the homologous gene *SlAucsia*-2 are highly expressed in flower buds and decrease dramatically (97%) after pollination/fertilization. Their suppression by RNA silencing causes parthenocarpic fruit development in tomato [14]. Furthermore, *Aucsia-*silenced tomato plants exhibit other alterations such as reduced polar auxin transport in roots and increased sensitivity to 1-naphthylphthalamic acid, an inhibitor of polar auxin transport. In *Arabidopsis thaliana Aucsia *genes [GenBank:AK224828, GenBank:AK224647] are annotated as components of the endomembrane system. This finding argues in favour of the hypothesis that also other genes here presented can be candidate genes playing a role in fruit set.

In conclusion, this dataset represents a starting tool for the study of genes involved in fruit set in tomato and in other crops bearing fleshy fruits and could contribute to develop new methods to confer parthenocarpy.

## Methods

### Plant Material

The two transgenes *DefH9-iaaM *and *DefH9-RI-iaaM *were introduced in UC82 tomato plants. The UC82 is a typical cultivar used by the processing industry. Two independent *DefH9-iaaM *parthenocarpic UC82 lines (#2 and # 3) and two independent *DefH9-RI-iaaM *parthenocarpic UC82 lines (#s5 and #s6) were used in this work (for a description of the lines see [12]).

### cDNA-AFLP analysis

For a detailed description of the method used, see [14].

### qRT-PCR Quantitative PCR Analysis

100 mg of pooled flower buds (collected from 4–5 plants) were ground in liquid nitrogen and total RNA was isolated. Starting from DNase-treated total RNA, first-strand cDNA was synthesized with oligo-dT primer and Superscript II (Invitrogen). The cDNA clones were amplified with gene-specific primers designed to give amplification products ranging from 100 to 150 bp, according to Applied Biosystem guidelines. Experiments were carried out using Platinum SYBR Green QPCR Supermix-UDG (Invitrogen) in ABI Prism 7000 Sequence Detection System (Applied Biosystems). The following cycling conditions were used: 2 min at 50°C, 2 min at 95°C, 40 cycles of 95°C for 30 sec, 56°C for 30 sec, 72°C for 30 sec and finally 72°C for 3 min. All quantitations were normalized to actin gene as endogenous control gene. Forward (F) and reverse (R) primers used for actin amplification are the following: F 5'-CCCGTTCAGCAGTGGTGGT-3' and R 5'-TACGAGGGTTATGCTTTGCC-3'. For each amplification reaction, the analysis of the product dissociation curve was performed to exclude the presence of non-specific amplification. Data from qRT-PCR experiments were analysed according to [29].

### Northern Blot Analysis

Total RNAs were isolated with Trizol reagent (Invitrogen) and then 20 μg were separated on 1% agarose-formaldehyde denaturing gels. The gels were blotted overnight on Hybond N^+ ^membrane (Amersham Biosciences) in 10× SSC. The DNA probes were labeled with [^32^P]CTP using "Ready to go DNA labeling beads (-dCTP)" (Amersham Biosciences). The membranes were hybridised overnight at 42°C in ULTRAhyb buffer (Ambion) in presence of 10^6 ^cpm mL^-1 ^of labelled probe. The membranes were washed 2 times in 2× SSC containing 0.1% SDS for 5 min and 2 times in 0.1× SSC containing 0.1% SDS for 15 min at 42°C. Autoradiography was then performed using Kodak X-AR5 film. For each c-DNA AFLP clone tested, the probe was obtained by amplification of cDNA with the same primers adopted for qRT-PCR.

## Competing interests

The authors declare that they have no competing interests.

## Authors' contributions

BM performed cDNA-AFLP, qRT-PCR and Northern blot analysis and drafted the manuscript; GLR is responsible for the production of the transgenic lines; TP carried out bioinformatic analysis and drafted the manuscript; AS conceived the study.

## Supplementary Material

Additional file 1 **Sequences of the 212 differentially expressed cDNA-AFLP clones**. A list of sequences corresponding to cDNA-AFLP clones differentially expressed in *iaaM*-parthenocarpic flower buds. Click here for file

Additional file 2 **Differentially expressed genes**. The differentially expressed cDNA-AFLP clones are listed with their (if available) accession number, e-value of the sequence that produced the highest identity score, and with the best *S. lycopersicum *Tentative Consensus (TC) sequence (DFCI Tomato Unique Gene Indices). Click here for file

Additional file 3 **Validation of cDNA-AFLP expression pattern by qRT-PCR in *iaaM*-parthenocarpic flower buds**. The data reported represent quantitative RT-PCR analysis of differentially expressed genes representative of the different functional categories performed using independent parthenocarpic lines different from those tested by cDNA-AFLP. Click here for file
