# Supplementary material for: Expression profile analysis of early fruit development in iaaM-parthenocarpic tomato plants
Source: BMC Res Notes. 2009 Jul 21;2:143. doi: 10.1186/1756-0500-2-143 (PMC2718906; doi:10.1186/1756-0500-2-143)
Supplement: Additional file 1 — Sequences of the 212 differentially expressed cDNA-AFLP clones. A list of sequences corresponding to cDNA-AFLP clones differentially expressed in iaaM-parthenocarpic flower buds. [file 1756-0500-2-143-S1.pdf]

Additional file 1

5
TATGGTCACAGGAGTTATACATCCTCGTGGACATGCCCCCAGGCAGCCCGAAGCGTGTCACCTTGTGGCCAGGAGTTATAGCCGATCTC
CATATCGTGGAAGATATGAAGTTCCTTACTCCAATGGAAATGGTGTCAGGGACC

6
GACACACACAATTACAGAGTAATCAGATACATACAGATTCCACACCCAACTAAAAGAAGTAGACTTTGACAATCACACAGATAAATCAT
GTATTAGAAGAATATCTTTGCAGGAGAAATTAGGCAGCACCCCTTGAGTTTCTTGGCGATGGCAGCGATGTATTTACCTTGGTGATGTG
CCTGCTGCAATTCAAGCTCAGATGGCTGTCTCGAGCCATCACCAGCGAATGTTCCAGCTCCATATGGACTTCCTCCTTTCACGTTTTCC
ATTTCAAACATGCCAGCACCGAATGTGTATCCGATGGGTACAAATATCATTCCATGGTGAACAAGCTGAGTTATAGCAGTCAACGGTGT
TGTTTCTTGTCCACCGCCTTGG

10
GCAAGTGGTCTTTGATTAGTCTTCATCGTTGTCATCCTGATTGTTTTTTTGATTGTGCTTCTGAGCGTTATACATATCCGCTACCATCT
CTGCTGATGAGGCCTCCTCAGGCTTCTTGTCTTCTCGTACACGGCTCAGACGTGGAAATCGTAATGAAATTCCCTTCGC

13
TCTCAAATTAGATTCTCAATGCGAAAGGTTTCTGGAGCTTGTGGTCATCTTTCAGTGGACCATCCATCCTTTGATGTTGAAAATCTGCA
AGGAAGTGAAATTGAGAACGACAAAAACAGGCCAACTCTAAGTTTGAAATGCCCCACAAATACTAATATTTCCTCTGTCAAATTTGCCA
GCTTTGGAAATCCTAATGGTACATGTGGCTCCTACATGCTAGGAGACTGCCACGATCAGAATTCTGCAGCACTGGTCGAAAAGGTTTGC
CTGAACCAAGATGAGTGTGCATTA

15
GCGAGTACCAGGCAAGTAACAGAGAAGAAAAACAGAGACACAAAGTCTCTAGCTGACCATTTTCGAACCACCTTAGCTTCAACCCTCAA
GCTCTTAGCCCTCCTAAGAGCATCAACTTCCTTCAACAAATCAAATTCCACCCCTTTTCTCTTCAGCTCCTCCACACACTTTGTCAGTA
ATTTCCCATCCTCTTTCTCTCTCTCCAACAGTTTCTCTAAGTGACTCTCTGTATCCGTCTTGTACCTAACAGCCCAAATAATACCCATA
GAACAGAGGAGAGAACAGATTGAC

16
TAACTCACAACAAGTACAAGAACAAACCTAGTGCGTTCCCACGAGTGGAGTCTGCGCGAGGATAGAGTGTACGTAGCAAATCACTTCCT
CTTATTCTACAATTAGCATTCACAACAATTCACCTCCACAAAACTTACATCAATTAATTACTCCTGCCGTTACTAATTATGTTTTGTTT
CGTTTTGTTTTTCCTTCTGTAGGCNTTACNTTCTCTATACTGTAATAGGTAGTTCTTACTACTACCGGTGCATGTTGAATTTTGAAATA
AGATAGATTTCTTGGTTTACTCAGGACTCATCA

29
TTTACTTTTCTTTAGGCATATGGCTTCTTCTGTACATGTTTNGCACTNTCTTTCNNCTCATNTGCCTNTNTCTATCCNCCTTTATGAGA
GTGCTGTGCATTTTCAATGGTGTTATNTTGNGAATTATGAATGTATTTTTGTATGGTTGGAGACTAAATATTCACTTGAGACTTGTTGT
ATTGGAGTTACACCCATCTTTTTGCCATTGTGCTTGAATCTGAATTGCAAGTTGTTGAGTGTTTACTCA

30
TGCTTTCTTTGATGGGTTACCACGGGATTGTTCAAAGCCGAAACCCTGANCATGTNNAGATTATCCATGGAATTGAAAGGGGTGATGCT
CTGCCNGGTTTGAGAACGTNCAACGGATATNGNTGAAATTGCTAANAAAGTGGGTCTTTGAAGTGGTGAAAGAGAAGGATTTATCANAT
CCCCCATCACATCCATGGTGGACAAGGCTNTAAAGCTCNCCCTTGTNTTGTGTCTACTCACGACTCATC

43
TTTTGGTGCCGTGAATTGCATTTTCAGTTGTTGAATTGTGATAGCTTTTTCATCTTTCGAACTCGAATCGGTAATCGAGGATGGAAATG
GAATACTCGTTGTTGATTCCATTGAATTGTTGAAATGAATATTTTTCGTGAGATTGATTTTATATGTAACAGAATGTTTACTCAGGACT
CATCA

47
CTGGGTNGCTTGACCCTTNGTTNTCATCTCCNTCTTATCTTATGAATAAATAAGGTAGTGCTTGATTCTATTTCATGCANTAATTAGTN
GTAATCGTTATGCCAGTAATTATCTAATTACGTACACTCTTGTGTTTACTCAGGGACTCATCAT

51
AAAAACAGGCCAAGCCCATTATGAAGGAATTGTAGATTTAGGACAGGTTTAGCGTAGGCCCAAGAATTTGGTTTTATTTCCTATCTTAT
CTTCATTTGCTTATTGAAGTTG

53
GCCTTAGGTGTTTGTAGCTCGGGTGTAAATCTGCTGGCTAGCATGAGCAGACACCATCCTGATCAAACCTTTAGCCATCAATTCCCTAA
TTGCCTTCCTGGCAA

67
GCGCCGGTGCNCAGTNTCGCANAANCTGAANCNGGCNATGNGGCCCNNATACCATGCCGCGAGGACTANNACNCTGACTNNANCGCCNT
TTCCTNCACTTCCCTTGTTGGGACTGAATGTGATNNTGNACATNCTGGACAANACACGNNNCNANTTNNTNTTTTGCATTACCTTGCAT
AATGNTGNNNCNTNCANGAACATCTNGNCTGCTGAGGATATGCCAGTTTCTTCNNGNNNNAACANCTNTCGCTACCNATTTAGCGATAA
TGATCATGAGTATGTTGAAGANACCACNCAATATCNCTTATGTGNNGTTCAAACCCATNANAATGNCTATGTNCTGGGTTGATGGAGCT
CAGATGANTCAAATGCAGGCANGCTNATTACCTTCNGNANNCTGNTCNTGTCNGCTGTGCTNTGGGCCATTNGGTGCCTCGCTCCATGC
GTGTCNCTCNNGACTCATCA

70
CGGAAAAATTGGATCGTCACCTGCATACCAAGCATTCACCAACGGATTCGTGGATAACTCAGCAATTTCATGAGCAATCACACTAATCA
TTCCGTCAACACCCACATCGTTATTCGGCCGATTTCACAGCTTTGNAACCCCGACCCATGTAATCNGGGTACCGGAAAACCGGGTATGC
ACAAACACTGGGGCATAGTTTTGCCNGGAGTTACCTACCCTGCGT

71
ATTTGTTCGCTTGGTGTCAGAGAATTTGNTGAAAATGATGAAGAGACGTCAGGTACCTTGAACATAAACCAGTGCAGAGAGGTTTGTAT
CCGAGTATGTCTGAATTCATATGTACAGAAGAAACAGCAAAACCCCTAGGGAATGTGCATCGGAGTACTTGATTTTGTTCTGCTAAATG
CTAGAACGCTCTCAGGGGATGTGCATTGTTACCGCTTACTTTGGTCTCCCATAATCTCATAGGTTGTAGCCTGTGATATCTATCTATCT
ATTATCTTATGAAGAAATCAACAGCTGTGTGCTCCCATAGTCTTGTAGTAGAAGATATCAATAGGTCTTATTGTAATTGTAATTTCATA
GGTGGAATGAAACATCAATTCAAGATATCATTTTTTGATAGATATTCAAGGTTACTCAGGACCTCATCA

72
AGTCGATAACTGTCTGTGCCTTGTAGAGAATTGAAGAANGACAGCTAAAAGCACTTCNTAGCCTTCTTCGTTCNGTAACATATTCTGGT
CCATTAGGGTCATGTTCCACCGCGGCAACTTTTANAGCCTCGGGTAGCATGCAGTTGAATGATGAACCTAATTTTGCAAGTCGGTTNAC
CCACTTTGGAATCTTTTTCCCGGTGAGCTNGTAGCATGTATCGTTGCAAAAATGGTTGCAGTTCTTCGAAATCAAATGATATGTGTCAC
CATTATAGCTAGCAGCCTTGAC

74
TTCTTTATAAATAATATTACTTCAGCTGTCTGTAGCAGTTGAAGGCATAGGCAACTGATATGGCAGCACCAACAACAGCAAGAACTGCA
TACGTATCTTCACGCTCCCAGCTCTCGTACCAAGTAGGCATTGAGCACACCTTGCGAGAACGAGTCCCTTTGCCACTGCTACTTCGAGA
AACTTGTCTCTTGAAGCTGCTCTTCTTTTCGGTTGTAAACCTCAGCGTTCTCTTGGTTGAGATTGAAATTGTCTGCATCAACCTGGTGG
TTTGAGTGGTCTGACAACATGAGACATTTCCATTTGCCTGGCG

75
GCCAGCCATTACAGGACGCATCGTNNAGCCAATGCTAGACCTTCGGCGACTGGNGGAATTACTGTCGTNTCTAGTNGGAGATACNNCCA
ATCTTCTCCTCCTCTTTTTAGCAGAACCTTCACTGCGCAAGCCCTGCATAGCAGCGTAGTAATTGCGTGGCCTCTAGACCCAGGCATGT
NTNTCTTTATCANAAAGCACGCCTGCTTTCTTTCCATGTCCTGCTCTACATCGTCTTTCTGCANTTGCTTTTTGCAATAAACCTGGCCT
ACCATTCTTAGATGACCACAAGGAATATGG

76
TTGACTACTTGGGCACCCAGCTTGGTCCATGCACAAAGCATCTTGGCCATCTGGGCTTGCCAAGTTGTACTCATGGGAGCCGNTNAAGG
TTACCGTATTGCTGGTGGNCCTCTTGGTGAGGTTGTCGACCCACTCTACCCTGGTGGCAGCATTCGACCCACTGGGTCTTGCTGAAGAC
CCAGAGGCATTTGCTGAGCTCAAGGTAAAGGAGATCAAGAACGGTAGACTTGCTATGTTCTCTATGTTCGGATTCTTTGTTCAAGCTAT
TGTTACCGGAAAGGGTCCATTGGAGAACCTTGCTGACCACCTTGCAGACCCCGTTACTCAGGACTCAT

80
TAGGACAAATGTATATAGGAACATTCAACAATATCCCGAGGCAACACAAGTTCCCGGTGTACTAATAGTGAGAGTTGATTCTGCTATAT
ACTTTT

81
ATCATGTCAAGTCTNTGGATGNGCNAGTATCTACTNNGNCATTGGGGCTNCCTCCTCANTGACATNATCCTTCTCGTNACATGTNTGAT
GCATGAGGTGTCTCTTGGTCTTACATTACATGCATCTNTGTGTGGAGGACTGGAAATGGTGGTGGAAATCCTTCTTTGCCTCCGGATCA
GTTGCTATATACATTNTNCTNTATNCCGCTACTNACGACTCATC

83
TGAGTTTAACACTGACAGTATAAAACCTTTTATGACAAAGACAACAACCTCGTATCCAGAAGACGAGATGAGCAATGAAGAATTCAAGA
AAACTGTTGAGAATTTCATTGCAAGACAACAAAGATTTTTGAGGGAGGAAGAGTTTTCAGCTGTTGTTTCTTATGAATCATAGCTTTTT
TTTGATATAGTTCATGATCATGTTCGTGTACTAGTTTTTTGCTCTCTTCTCGTAGCAGACAATCTATCTCAATGCAAGCAAACAACTGA
TGTTTATGTTACTCAGGACTCATCA

84
TAGATGCCCTCAGAGGTAGTGGTTTCATAGTATACGTTTTGCCACTTCCTGTTTGTCCATATGCAAAGCAAGTAGCTTTTGTGCGTTGG
AAAATTATTGGGACAATGGGCTCCACCGTTTCATGGTATACCTCATCATTTGAAACCTCCTCATTTAG

85
ATTTTTNTTCGCCGCTTTTGGATTTTTATCCCTCTGCGGNTTCGGCGCGGTAGTAACTTGGGTGTACAAGTACTTCATCAGCAGGAGAT
CGAGATCAATGGAAGAAAATTATGATCATAGTACTGATACCGCTGATGTGAAGGATTACGGTAGAGAATTTGGTGGGTACCTTCAGTAT

AAAGAAAAGGATACAGCTCCCGGTGCGTAATTTGGGGGAAAACGGGTGGGGATTGCGAGATGAGAAATCGAAAGATTCAGATAGTTACT
CAGGACTCATCA

91
CCGGGTTGACTCCTTCAAGGAAGTTTCGACCCGATCAATTTTCTTCGTTTTCGACTTCTTAGTGAGTACAGACGGAACCGGTTCCTGAG
TCTTCATAGTTTCGAGCTTTTTTGAAGCCTTATCGATTGTAGATTTGAGGGATTTGTATGAAGTAGTGCGGTATATAAGGAGATATGAA
ATTGCCTCACATACTATAGCTGTGCAGATTGAAATTCCGACGAGTG

92
CCCCTGTGTTGGTTGTGGTTGTGGATGGTGTTTGTGTGGTGGAGCCCGTAGTCGTCGGAGTGGTGCCACTTGCAGTAGCAGGATAAACA
CAGCCAGCTGTGCTGGGGTCAGTAGCAGTGACAGTGGCGGTGCCAGAAAAATCACATGATCCGGTAGCTTGGCCCTTCCTCTGGAAATA
GCTGTTGACAGCAAAATTGCAGTGAGCTCTTACGGTATTCG

104
CCTTTCACTTTTCAATCGGGGCTGCTAATTATTATTACTCCTAAATCTGACATGATTGTGTTAGAGTTTGCTCTATCTACCACTGTTTG
TACTTCAGAGGAAGTAATTTTGTCCACGTAAATCGTACTTTTTGCGGAAACAGAGGATGTTCAGACATACCA

109
CTTCCATAAGCGAGTCCAAAGGTAAATGCTGATGCGCGCGCCACCAGAGCAAGAGTGCTGGTTCCTGAGTATGGTGCTGGAAGAGGTGC
CATTGTCGACTACAAATTGAGGAACATTGG

112
CTTATGACTTGCTCAACATGCTTGCTCCATCCTCACAACGGAAAGACGCTCTCGAGGGTTTTGTAAAGCCTCACGGAGANACTGATCCA
CAGTGTTACTCAGGACTCATCAT

117
TTTTCCTTCCCAATTATTTCTTTAGCAAGCTTTGTTGGTACACCAATAAGTTCTGGCCAGAATTGTTTTCCTTTGCACCAAGATTCGGA
TTCAAATTCCTTTAGAAGTTTTAAAACCACCACGGAGACGGAGGACAAGGTGCAGGGTGGACTCCTTCTGGATATTGTAGTCAGCAAGG
GTGCGGCCATCCTCAAGCTGCTTACCAGCGAAAATCAACCTCTGCTGGTCTGGT

124
CATGAATCCTNCTGATATTCAAAACATACACAAAAAAAAAAGGCGATAAAATAGTTGAAACAAAAAATGTGTTTACCATAAAGCTGCATT
TCTAACAGCCTACATCTATCAGACAAAAATGAGGGAGTACACCATACTACTTGCAAAAACAGTATCATACCACTAGTCGAATATACAGT
AATGTGCATACAAGATGGGGGG

125
AGGTCAAGTTTGTGTATCCAAAGAATAAGGATAGCGCGGAACTGATGAAGTCGTACTTCGACGTTGACAATCTTCCTACTGAGTTTGGA
GGAACAGCTACACTGAAATATGATCACGAGGAGTTCTCAAGGCAAATGGCTCAAGATGATGTGAAGGCCGCCAAGTTTTGGGGTTCGGA
CAAACATCCCCCTGGTCCTGGTGGTAATGGTAATTACTCTGCAGTAGAAGTTGCTCCAGAACCTTACTC

126
TTTATCTATCGCTTGNTCCTCTTGTCTGAGATGAGCATACCATCTATTGAATTACAATGAGTACAATGAGGTTATCACAAGAATCTGCA
TAGATGTTTCAGNTTGTACCTGCAAATCTCAGGCTTCTTGTGTAACAACAGATTTCAGCAGCTTTGTAATAAGCTGAAGTATAAATTTA
CAACTCTCAAGATACATGGTGTATTATATTTGCATGTCTCAAGGGACAAGAAATCAATGTTTACTTACTCAGGACTCATCACTCAT

128
TAGAGCTGGCTACGACGATNGNTTCCGAACACACAATGCGATTAAGCATTCAAATCCAANANATCCTTTTCCATTNGCTTCCTTANTCA
NGTACTCAT

131
GCTGCAAAANTCAAGTGTATGAATTTTCAACATAGATGATNGANCTCTATACTTGCAANCAATATTTATACATTNTCCTCAACTAAGTG
AATCACACATGGNTCCATTAGTATATATACTNAGCATGATATCTTTGTGATGCATNTTTCAAACGG

132
TACCCTGATCCNCTCATCAANGCGCAATNACACTATCAAGCTTNACTTAGATTCGAACAAGANTGTTGACTTCATCAAGTNTGNTGTAG
GNAATGTGGNCATCGGTTACTGGNGGTAGAAACATGGGGACCNGTTGGAATTCTNACTCAGGCACCTCATCA

133
CCTTTCTTTTTACTTAGATTGTGAGCTGAAATAAGTTCATGTAACTTGCGCATCATCATTTCTTCCTCAACCTGAAGCCTGTGCTATTC
ATCTGACATTGCTTTATGGGCATTCAAGAGTAGTTCACTTTCTTTCTCTAGATGCTTCAAAGTTTCTCCTGCATATTTGG

134
TATACAAGATACCTACACCATGAAGNCCAGCNGGTTAGTCGAAACTTATCATCATCACTTTTGATATCTTAACCAGAGCTTCATCTGTC
TCTTTCATTGGAGTTCCATTTGACTCTGCTATTGGAGCTTCCTTTATTATCTCCTTACTCAGGACTCATCAC

136

GACTGGGTCAGCTTGNTTCAAGCCTTTTTTATTTGTTTCTTCTAGCGAACTGTACAAAGGAATCTGGGATAGTCCTTGGATAAAACCTC
TCTCTCATTTTTCTATTGCATATTGAAAACTAATTGGCTCCACTGAGCTTACTCANGACTCATCAAT

137
GATTCTGGAACCAANGCCTGCAAGATCGTAGTTCCGCACATGGCATAGAGTATGTGTTGATATGCGTTNNTTGGCGANCAAGCCCATAT
TANTAGTGCATCAATTGCTGAATGGTTGTGTNTATACAAAACAACGCTCACTAAGNACTCATC

140
TTTCGCTNCCCTTGTAAGNGCTATGTTTGNTTCATGTGGACATTCCTNTGGTGGTGCTCATGCTGAAAAGGGTTATNAGATATGGCAAG
ATGTATCCTCTGCAATGCTGAAATTGAGNGTGGTTCCTTACTCATGNACTCATCA

143
AGCACACTCTGGGGGCTTTTTCCTGGTAGCTGGTAAAAACTNTGAACCATGGCAACCGGCGAAGGTCATGAAGAAGGTCAAAAAGNTCA
GTTCACCANTACTTTTGCCCTTACTCAGGGACTCAT

147
GCCATCAAAGAGAAAAATGAACTACCCATGAGTCTGATGCAGGAGGCATCCCTTGATCAACCGTGAGCTTCTCAAAAGCCTTGATGATG
TAACCTTTGA

149
AATTATGTACAAGGGTGATCAAAACAAAGNAGTGCTGAATTCTTGCCAAAGACGCTNTCCATAGCATGCTNCCTTACTCAGGGACTCAT
CAT

152
CCTCTCTATAAATAGAAGTCGATTCTCTCAAAGCTCTACACGCTTCACTTCCTCTCTTAGCTTCACACAAAATTACTACAATGGCTGGG
AAAATGAGCATTGTCTTGTTTGTTCTTTTGGTGGTTTTCCTGACTCAAAATCAGGTTTCAAGGGCCAACATTATGCGTGATGAGCAGCA
GCAACAACAGAGAAGTAACCAACTGTATGGTGTTAGTGAGGGAAGACTCCATCCTCAAGATTGTCAACCAAAATGCACGTACCGGTGTT
CAAAGACATCGTATAAGAAGCCGTGTATGTTTTTCTGCCAGAAATGCTGTGCAAAGTGTTTGTGTGTTCCTGCTGGTACCTATGGCAAT
AAGC

153
TTAAAGCTCTTATTTCAGTGGAAATTACTAGAGGTAGTATCAGCAGCAGCATAGATTTGATATATCCTTAATGCTTAATTAGCTTCTGT
TTTGAAGGGCTCCAATGAAGCGTGGTACACTGATTTCTTGCTGAGGTAATGTGGCATAAAATCGATTCCCCAATGGAAGGGTTTGAACT
GTGGAGGATTCTCAGGACCAACAAGTGCTTTTGCAGGTTCAACAATCTCATGTGGGCAAATAAAAGTACCAATGGAGGTTCGTGACTCA
GTTGTGTTTGTCACCACACGATGTGCAACA

155
AGCCATTCGCGCATCACAATTACCGAGGTCATTGAGCATGAATGGTTCAAGTAAAGGGTATTGTCCACCTGTTTTTGAACATGCAGATG
TTAGTCTTGATGACGTGAATGCTATTTTTGATGAATCTGCTAACTCTTCGAATCTTGNTGTTGAGAGGCGGGAAGTACGGCCTGCTGCA
CCGCTGACTATGAATGCTTTTGAGCTTATTTCAACTTCTCAGGGTCTCAATCTCAGTTCTCTGTTTGAAAAGCAAATGGGGCTGGTCAA
AAGGGA

156
CAAAAGTATTGCAGTTGAGTGCTACCTCGTCACTTTTGCCATGTAGTATCCTGCTTATGTGCAAACTTATTGAGAAGTAACAAACAGTC
CGTGTACCACAAATATATTTGCATGTAATAATCTCATCTTTTTTAATATTACTACCATTTGCTTTCACTACAAATGCAATAGGAACNTT
CTCCAGCTACTTCATCTTTCATCGGTACAACAGCAGCATCAATAATATTTGGATGAGAAATAAGCATAGCTTCTAATTCAGCAGGAGCC
ACTTGATATCCTTTGTATTTTATTACTCAGGACTCATCA

159
CTCCTAGCATCTGTTAGTGAACATCTGTTAGGGTTTGGACATTGAGATCGGGTAGTGAAGGTGACTGTCTACATGAGCTTAGTTCAAAT
GGCAACAAATTCCACTCGTGTGTTTTCCACCCTGCGTATTCGTCTTTGCTAGTTATCGGCTGTTACCAGTCTTTGGAGCTATGGAACAT
GAATGAGAACAAGACAATGACTCTCACAGGCCATGAAGGATTACTCAGGACTCATCA

161
TGACTTCATGCAGAAAACAAACAACTACGATGGAATTTCAGCCATAAGCATAGGGGAAGGGCTTGGGTTCGGCCAAAGGAAATCGTTGC
AGAGGAAAAGATTGGAACACTGTAAACTTTTAGTGAGCTCTTTTTGTTCTTTCTCCTATAGGATTGAGTTGTATATTACTGCACGACTT
GTTACGTATAAACTATGCCTGTTTGGGGTTAGA

162
TTTCTGGGTTGCAATTTTGGTAGTAGTAGCAATTAGTTCTCACACAAATCGTTGTATTGGGCGATGCTATATGAAGTAAATCATTCCTT
TGGATATCCCATATTTTAGGGTGGCAAAGCGTATAGTTTCTTGCCAAACCAGAGTGGATAGTTGTATCGAATATATGATGATGAGATGA
AATGGTGTTGAGCTGCGTTTGTTGGATGTATTACTCA

163

CTTGATGGTCTGAGGCTCAGCCTTTGAGTCAGATTCAGCAATTCTTTGCTTTATATCCCGGGCTATTGAAAAGAAAGCCTCCTGAACAT
TCATATTCGAAGCCTTAGGTGTTTGTAGCTCGGGTGTAAATCTGCTGGCTAGCATGAGCAGACACCATCCTGATCAAACCTTTAGCCAT
CAATTCCCTAATTGCCTTCCTGGCAA

164
TTGATTCAAAGAGACAGACATAAGAACAGAACCATCTCGATGCAACACGCCTCTGTAGTCTACTTCATCACCCTCCTTTTGAGCTGGCA
ATTCCCCTGACCTACGTTGAAAGTCAAAATAACGTCTGTGCTTTTCTTCAAAGGGGGCCACTCCTTGCTGAAGCGCAGCTGCTCTTTTA
CCAAGGTCTGCCAATCGTCGACA

165
ATGGCACAAAAATTTACTATCCTTTTCACCATTCTCCTTGTGGTTATTGCTGCTCAAGATGTGATGGCACAAGATGCAACTCTGACGAA
ACTTTTTCAGCAATATGATCCAGTTTGTCACAAACCTTGCTCAACACAAGACGATTGTTCTGGTGGTACGTTCTGTCAGGCCTGTTGGA
GGTTCGCGGGGACATGTGGGCCCTATGTTGGGCGCGCCATGGCCATAGGCGTGTGATTACAATTTCGTTGTTCTTCTTTTTCGACTTTT
TAATCCCAAGTGAATAAAGTCTAATTCGAAAAAGAAGAAAAAAGTATCTATGTCTGAGTTATATGTTTTGTGGCTAATAAGAAATCGAC
TATGCTTGTTGATTTGATAAAAATTATGTCATTAGGGTGTGATATGTAATC

174
AAGATACTGAGTTATTGATTAGGAAGCTACCTTTTCAGAGACTGGTTAGAGAAATTGCACAGGATTTCAAGACGGATTTGAGATTCCAG
AGTAGTGCTGTAGCTGCTCTTCAGGAAGCGGCTGAGGCGTATTTGGTGGGCCTCTTTGAAGATACTAATTTGTGTGCAATTCATGCTAA
AAGAGTTACGATTATGCCTAAGGATATGCAATTAGCTAGACGTATTAGAGGTGAAAGGGCTCAAGAAGGGAGCTGATGATGGTTTGACT
CCTGAGCAGCGCCGTGAAAGGGATGCGAAAGCTCTGCAAGAGAAGGCTGCAAAGAAAGCAGCAAAAGAGGCTGCCGGAGGTAGCAATGC
TGGAACTAAGGATACCAAGAAATAGAAAGCTAGGTGTTGTGTACTTCATTGCAGTCAGTAATTTGTCATTCCCCTTTGTTTGTCATGTT
ATTCACTGTTTACTCAGACCTCATTCA

176
GTCCATTGNCCATGTTTCATCNAGCGAATACGCTCNCCANGAGGTGATCTNCATCATCTCCACTGNGCTTGGACTGAGACGTGACTGTN
GGGCGCGTNATGCTCTACTNTNTAGGATGCTATNGCGGNGTAACATGGTCTTATCANTTGCCAAAGATATAG

179
TTTGATTTTGTTAGNCCAACTTTAGCAATGGCATCGTTGAATCCATATTGGAGCAATTTAGGTCCCTCAATAAAAATGGCAGGCGGGAG
GCAAAAGATAAGAGCAATGATGGATATGTAGGCATACACGTTAGTACTATCCATATCAGTCATAGCTTTCTTCGAGTATATACTCCTGT
ATGTGAAGGAGATGTTAGAAATCATAGCACTAGTGAAACCCAACCAATTGAAAGATAGCTCAGTCAATGAAGCCATCGACACACCAAGG
ACAACTGGAGCAAGTGACAGCCATAGTGCTAAAGGTATTTGTTGTCCAAGAATGAACTGAGATGCAGCAGCATTGAAGAAAGGTTCCAG
AGCTTTACTCAGACCTTCATTCA

180
TTGGGAGAAAGCTCCNTACAANCAGGANGCTATTGTGCTTAGAGGACATTCAATTGAATGCCGTATAAACGCAGAAGATGCTTTCAAAA
ACTTCAGACCCGGCCCAGGGAGAATCACTTCATATTTACCAGCTGGAGGTCCATTTACGCGTATGGATAGCCATGTTTATCCTGACTAT
GTGGTTCCACCTAGCTACGATTCCCTGCTAGGAAAGCTCATCGNATGGGCTNCAACACGCNAGAGGGCTATTGAGCGCATGAAAAGAGC
ACTTGATGACACCGTTATCACTGGAGTTCCTACCACAATAGAATATCATAAACTCATCCTTGATATCGAGGACTTTACTCAAGACNTCA
TC

187
TAAAGCGTAAAAGTCTTTTTCCTCCCCTTGATAGAAGAATGAAGGGAGGAAGTCAATTGCCCCTTCTCCAGCTAACCGATAGGGAAATC
CTTACACAAGCCCAAAAAAAAGCATGAACTCGATCTTCAGCCCTTACCACGATTCTCTCAAAAAAAGGAAGGAGATGAATCTGCCTAAG
TAAGGAAGCTGGGGCTTAGTGTGAGTCACTTCACTCGGGAAAGAACAAAGAAAGGGCAATCAATTGTTTACTCAGGACTCATCA

192
TTTGCTGGTTGTTCTGGTGGTGTGGCGGGCCTTCGTGTTGCTAAGGACATTGCTGAGAATAATCCAGGAAGCAGAGTCCTGTTAGCTAC
TTCAGAGACTACTATAATTGGGTTCAAACCACCTAGTGTTGATAGGCCATATGATTTAGTTGGTGTTGCTCTTTTTGGTGATGGTGCTG
GAGCTATGATAAT

193
TTATTCAGATNCATCTGCATTGGNTNCTGGGGGANAGATCATGATNGGGGATGGAANAAATTAATGGAGCTNGACGGANATGATGATNG
ATNCNCNGNTNCTGATACCCTNATTANNNNAGNNGGTNTANAAGTGNTCATTGGAGAGAGCANANCGTGCTTTCCATNGCNTNGACTCN
NANTNTACGAGAGAAATTGNATGGGGNGTGATTNACTCANGAACTCATT

201
TGGGNTTGAGTACAACATNAGCTAGCAATAAATTNCTCTGTAGTCATGTGTGATTNCTAAATAGCTCCTCTGATGATTGACGCGGTTCT
CTCTATCAGTGTCTGNATTTTGGGTGTCCCATGTTGAGNATNCATATCAATTGANTCTATTCTTANNGACTNCATTTACGTCANGACTC
ATACATCATCA

202
TACGTGGCTGCTACAGNATNGAATNTTCCAAGCATTCGCTTCTGAAAGAATTNGANCTAATTGTGCCACAGTTGAGCGTNTCTGTCCAA
TCGCTACATAGACACAATACAATGTCTCACTCTCAGAGGTGGCCCTTGAGTTCANTTGCTTTTGGTTTACTCAGGACTCATCATCAGA

204
TCCACTTGGGAGCCNTCAATGGGTTCATCCGGNTNCGCCTTGCTCGNTGACCGGAAATATCGCGACATAAGAGGTCNCTGNTTTATAGT
GANCTGTNNCTGCTGCTGCTGGCCNATGTAAGCTGCTTGCGTGGNCCTTTACTCAAGACTCATACACATCAT

208
CGAGCCGGNTCTGGNTGNNATCTNCAGTACCGGGTAGCCNGCCGGGTTCTGATAGCGNTTNGAGCCTTCNTGNACCGTCGTCGGGCACN
NTCTNANGTGTTCACNCAGANNCNCATCACA

209
CTAGTTGTATNANGGGTGCTANCTTCNGTACAANAATATANTGCTTNGGTAATGNTAGTCNNATTCGGCTCCTGATGACACACCTTGGG
C

212
ACAGGCTGACAATCTAATGCTTCCTCAAGTTATATACCATGGGTGCCAATTAGGAGTAGCCNTCACTCTCTTTNACCAGGCTTGTTATG
TGGTCTAGCATAATCTCCCACCACACGTTGCCAAGACATTCAGACAGCTGAANATATTGCAGTCCGTAAAAGCTTATATATGAGCATGA
GAATATATCAAAAAGAGAATCTCTAGCTTGTGNTTGAGGCTATGTAAACCACCCTCTTCATGCCTTACGTGTGAAATGTCATTGNGCAT
GTTGCTGGCTTTGAACACAGNATATGCTAGTGGCGAGTAAGTTGANCAAGTGTGCCATTNGAGAAGACTTCTCATATCATTTCCTCTTT
CCTAGCCAAGTGTTGACAATAATGATATTNGATATAATTACCCCTTTTGATATAAATTTATCTTTACGTTACTCAGGACCTC

213
TTAGATGNCTACGATGCTGACGTNTGCNCCATGCNGGATCACTCGTAAGCTCNAGCAGGCAGNATACTANGNNCCATCATCTGAGACTC
CGGAAGTCAGGATAGTNGATAATGCTGCNANTGNTNCTGNAGAAAGTNCTGNTCCGCTCGCT

216
TGGTGGCCGGAGGTAAATCCGCCGCCGATGTCGAGTACCGTCATTTTTGGCATTCCGAGTTGAGCTGCTGTTTCGAAAACCTGTTTCGC
TGCGGCTATGGCTCCTAGATAAGCGTTGGAATCGGCGTCTCCGCTGCCGATATGGAAGGACACGCCGGAGACGGTGAGTCTAGCGGCTT
GAGCTGTACGGAGGAGCGGCTCGATTTCCTCCGGTAATGCGCCGTATTTTGGACCCATTGGGCATCTCGCNGTTGCCGTCGGTCATGGG
TTTGATTCGAAGCAAAAGCTCGCATTTTGGGGTGATGTTTA

221
TCGTACTCCAATACCGACATTGTTTCCATCGACAAGGCTAACATCGTAAAAGTCCTTCTCAGCGTTGGTTTTCGCGATTGTGAATTCAA
CTAAACTCACCGGAGGTGCGCCGCCCGCTCCGCTAGGGCATTTCAATGCACCGCAATCGCCGGTTATACATTTACCAGCTCCGGTGCTG
TTGAAATTGCAACCGGTTCTTCCCCAGAATCGGCCGGAAAATCCACCAGGAGCGGAGAGTTGGATAGTAGCGCCGGGAGTTACTCAGGA
CTCATCA

225
GTCCACGTCCAAAATCTGCATTCGTCAGTCAATAGCGAAAGCACTTGTTGCNTTTTACCAGAAGTATGTTGATGAGCANTCGAAGAANG
AGATCTGAAAATGGTGGCAGCTACCATGATACACAACTACAATATTCAATTAGTGGAAGCACAANCAATTTCACCAACAGCTACTGTTG
TGATTC

227
GAAAAGCAAAGCTCCAACAACCCTCATAATATCCTTGGTCCGGTCACGGATTGAGCTCGTCACCGACGAGTAAAGTTCCACCGGAATCA
TCGACGATGATCGGCTCTCCAGTTCCTCTCCATCTTCCACATGTTCAACAGCGTCCTCTTGTTCGAAGAAAACCGGGTCGGGTCCACGG
ATTAGAAATGGGTCG

233
TGGTGGGACNCATTGTCTATTCAAGAATTTTGACNAGGGCANAACTNNTNTNGNGCTNTCTNTCTTNTGNNANNCNACTCACAAAANCT
TGTACNCAATNAANCAGACGTTTNACCAACTNTACGNAGGACGGTTGAACCANATNATCATGTATACTGTTNCGTCACGTACTGATCAC
ATCANCGCNG

235
TCCGCTCTCGCCCAAACGTCATNGTAGCCGTCTTCATCGNCCGATAGCTATTCTTATTCCTCATCATCCGAAGATTACAGGTCTAGTTA
TATATAATTCTTTTCTTATGGAGAATGATTTTAGGGATTTGACGATCTTGCTTTATGAAGTTAGGGTTACTCANGACTCATCAT

237
AGAGTGCAGTGTGTATAGAGTAAGAAGTAATTGAAGGAAATAATAAAAGCCTTGAACTGTCAAGTATTCAATGAAAAAGTCCAAGTACA
ATCAGCCAGAGCTCTACTTATACTAGGGAGTTGTTTTTCCTATGCTGGAGAGCCAGTAGTGGAGCAATGTCTGTTACTCANGGACTCAT
CA

238
TAGGCNTTGTCTCAAATCTGGTGGTATGTCTAATGAATNATACAATTCAATTGCTCGAGTCACTGATGGAATTTATGAAGGAATTGCAA
TCGGTGGAGATGTTTTCCCTGGCTCTACCCTTTCTGATCACGTTTTGCGCTTCAACAATATCCCACAGGTTACTCANGGACTCATCAT
240GGGGCNTATATAAGTCCGGTCTAGTTTGCTAGCACNTCTAGCATGCAGTTCTGATCAATAAAGCTTGTGAGTATAGCATGGATACA
TACATGGAAAATAGTCTTTTATGTATTAATCTTTCCATCTGATCCAACATCAACCTCTCGG

240

GGGGCNTATATAAGTCCGGTCTAGTTTGCTAGCACNTCTAGCATGCAGTTCTGATCAATAAAGCTTGTGAGTATAGCATGGATACATAC
ATGGAAAATAGTCTTTTATGTATTAATCTTTCCATCTGATCCAACATCAACCTCTCGG

242
TTAACATCACCACTAGAAAGTCTCCTTGTGGAGAAGGTACAAATACATGGGACAGATTTGAGCTTCGTGTCCACAAGCGGGTGATTGAC
CTTTTCAGCTCACCAGATGTTGTGAAACAAATCACTTCAATCACCATTGAACCCGGTGTTGAGGTTGAGGTCACTATTGCTGAATCTTA

256
GCCTGCTTTACTTCTGAAGAGTATATGTAAAGGTTTTTGATCTTCAAGACAAGGTTACTCAGGACTCATCA

257
CTGATGCCTTTCCAGTTCAGCCACCGGCATTGCTGGAGACCTAATATCTAAGATCACAATCTTGTTGCTATCCATCAATATGGTAGCCA
TGTATCTCAAATCCTGTTTGTTCCAAGCCAACCTCAACAATGGCGTATCCGGTTTCGGGCTCTCATAAATAATCGTCGAGTGTTCCTTA
TCTCTCAAATCAAAAATCCTAACGGATC

260
CGACACACACTCATCGATCTTATTGAAGAGTCCGAGCAATATAGCTCTGTACACATGGAAAATCATGTATCTTATGTCTTTTTGATAGA
ACATCAACACAAAGGAAATCTTTTGTGTAAACAGAGTTATTTTATTTTCAACCTTGTAAGATCATCAGATAACTTGTTTTGCTACCTTC
ACAGCCTAGTAAATGCTCTTGTTTTGTTTACTCAGGACTCATCACTTATGCATGAATGTTCGTCTCCGCGTGAACTAAAGGTCATTTTC
TGCCA

261
TCAGCGATAGCAATTGCCTCAGGTGATCCAAGTAATCACCATCAAGCTGCATTATCAATGTATCCATCAACAATTCCTGGCTATGGATT
TGCCTCTGAAGTAGGCGGATCCTCTGGGTTTCGTATTCCTGCTCGAATCCAAGGTGAAGAAGAGGAGCACGATGGCATTTCCGATAAGC
CATCCTCTGCTTCCTCTGATTCTCGCCATTGAATTAGCTCCAAAAAGTTTGATCTTTCTCAATCCCTGACAGG

265
TGGAAGTGGNGTCCACGTCCAATCTATGCAATTCGTCAGTCAATAGCGAAAGCACTTGTTGCATTTTACCAGAAGTATGTTGATGAGCA
GTCGAAGAAGGAGATTAATTACTCATGCAAAGTGTAATTTTTTACCTTATGGAAAAGATTTCATGGGTGGTAATAATCCAACTGGAAGA
TTTAGCAATGCCAGGACACCAGCAGACATGTTAGTTGAAGATTTTGGGATTACTCAGGACTCATCA

269
GTCGCTGTGTAGGTCNGCGNGGTCTGATAACTCCATCCTATAAATTGNCNCTNGTNGAGNNGAGGAATTGTCTATGTTTGTGTGATAGN
AACCATAGTAAGTATATGGATATATGGTGGATGGAAGAGTATGGAATAACATGAATCTNGGATNCTCANGACTCATCCATC

270
ACTTGCCAACCCCTTGTTGATCAGCTCCTTACTCAGGTTGAAGCACAGGAGGCTGTAATTGGCAACATATCAAAACTTTATGATGTTGT
TGAAGCTGTCT

273
AGGTTGGAGGGCAAAGTAGCTATAATTACCGGTGCTGCTAGTGGCATTGGAGAAGCAAGTGCTAGATTGTTCGTTGAACATGGTGCTCG
TGTCGTCGTCGCCGATATTCAAGATGAACTTGGTCAAAAAGTAGTTGATTCTAT

278
GACACAATGCAGGTACAATGCTAAGCGTAGAACACTGGCGTAGGACCAAGCTAGGATTTTAATAAAATGCTATCTGGCTTATCTAGCTG
CCACACTTTTGCTTGTTATATTTAGTTGGCTGCTGGTGTATACAATAGATCAGGTATTACTCAGGACTCATCA

279
TTCGCTGTTGCAGGNAGTGGTTACTTTGNTGTCCGCGATGTGAATGAGAGCTGGATTCGCGTCTGGGTAAAGAAAGGTGGAATGATTGT
TCTTCCTGCTGGAATCTATCACCGCTTCACGCTTGATTCAAGCAACTACATTACTCAGGGACTCATCA

283
ACTTATAAATCTGCNATATTGGTACTATTTTCATCNNAATCAAGCTCGTTGGATCACGCCATTGTTGAAGAGAACAACTCCTTTNTGAA
GCTCATCAATGGCTACGTACAAATACATTACTCAGGCACTCATCA

284
TAATAACACAACATCTAATAATCCCAGGTCACAAAATTTTGCTCACAGTTTTATGCATAAATAAAATTTCAAAGTCAATAGTAATAATC
TATATCTCCTACTAGAGTAATGTTGTTCGCATCG

303
TCTTATTTGCATTTTCCATACATTCTTGTTGAACCTTAGCAATCCTTTGATTCCTCTTCCTTGACCATGCTTCTCTCTCTGCTTCAACA
CTTTGATAATCTCTCACCCAACAACTCTGCACAGCACATGTCACAACCAACACTCCTATTTGCATCACAAACATACACATCAAAACCCC
ACATTCCAATCTCACTAAAACCTTTGCTTCTCT

306

AGACTTGTTTCACTGGCANGCTNCAATAATGGGGCCCTNTNATAGCCCTNANGCTGGGGGTGTCATTTTCGGNCACGGATCCATTTGNC
TGCGGATTATCCATTCANGCCTNCTAAGGTAGCTNTNAGGACAAAAGATTGNTNCATNCACATATNAATAGCNATGNGAGTNTATNCCA
TGGACATACTGAAGGAGCATGTGGANCCCTGCATTACTCATGAACTACATTCATTAAGG

309
CCTTNTCTGCGGTATGTGCNAANCTNNATTCTGCNCACATCTCAGNGCAAGGATATAGATGNGCCCACAAGTTCANTNCAACGCATGAG
AATGTGCTCCTGACNGGCCTGGACTATCATCCATGCAATANCTATNAGTGGAGTTCTTGCNCTTGNTACTTTGGACTTTNCNNAANCGT
CCGNAGTACTGGTGATTACTTCAGGACTCATCA

314
GCAGCCAGCAATAGGGAGTAGTGGCATCACTGGATAAGTTGAAGGAAGNTGTNCACCAAAGTTCACAATGCAGANAGACTCACAAGCTT
CTGGTCTCCAAAATTCTTTCTTTGCTGCTGGTNTATCACCTTCTGCAACAGCNGCTNACAAGACACCAATGCCTATAGAAGAAGCCTGA
GACCCTTATTACTCAGGACTCATCA

318
GTAGTGTACCAGCAGTATCCTGAAGCAAACTTTGTGGAGTCAAACAAATTCNACTACGCCATCGTTGTANTAGGTGAAGTCCCATATGC
AGAGATGATGGGCGACAGCTCAAATCTTACAATAACAGAACCTGGTCCTAGCACTATTACTCAGGACTCATCA

321
TTTGCAGCAAGCNCTTACTCGATGGTATNGTGGCAGCTTCCTCAGGCATTGATTGCTGATCGATGGGATAAGAGTGAATTATGAACTGA
TTATTCCATTGATGGAGGTTATGTTCTGATTTTGTTCTTATATTACTCAGGACTCAT

323
CGTGATCGAGATTTTTGGTAGTGCACTTGGGTTGTTTGGTGTTATTGTGGGAATTATCATGTCAGCTCAAGCATCTTGGCCATCCAAGG
GTGCATAAGCCTTCACATTATGTGCTTGTTA

331
CCATTCACTTGTTACTTTTCTATCTTGTTATTTTATCCTGATCAATTTTTGAGTATTCAGTCGATTATGAGATTTCTAGCAGTGGATGG
TGGACTGATATCTTCTATTAAAAAAATCATGTAATCCTGTTCTCTTTGTATTTCGAGACAAGTTGATTTAGTAAAACGTTGGATTAATC
GACCATAAGCCTCTAGACATGCATAAGAATATTCTGACATGACTCGGAATAGTTCAGGCAACCG

340
GGAGGATGGAAGGGAGGGTAGGGTCTTTGCCTGGCCACTTGAGGAGAATTAGAGGTTTGCCCTTGACGAACTCAAGGGTCTGGGACTCG
AGGGATAGTAACTTGGCCTGTGATATTATAAATTCGGCAGCTTCATAGTAGTTGGGGTGAGGTTGGGCTGTATTGATCTGAAGGTACTG
CGGGAATCTTGATAGGATTGTTGA

342
GGNTTGNAANCGNCNGAGCNCTNGATNGGCAAGCTGCCGTCTCCNGCGGNNGGCNCGNGANATCGCTNAAGATTGNNAATGACCANAAT
GACCCCATNATACTACATGCTGNTTNCATGAANTANGCTCNTGNNGGTNTAANCNACGACNTTACCATGNCCACCAACAAGCGGAGCNA
ATNNTGGCGCCNCCNTNCTCANGANNCATNATA

344
CGATCAGTCCCTCTGTATGATAAGAAGGGTCATAAGATGAAATCTGTTAGACCTCTCATGCGATTTCAATTTCTTTGAACTCCAAAGTT
A

354
CTAGGCTTGTTCGNTCCCGTATATANTCGTGTATNCAGGNTAGTATATCCNGCCTGTTTTACAACTTTCTGAATTTGAAAACTCCTTNT
CANGANTGTAATTTCNCTGTCNNTAACTCAGATTTCNAACTCCGATCCNCNTG

355
TATGTAAATCAACAAGCGGAAGTTTATCGTGCCAATCTATTAGCAGTTTTGAAAGACATGGAGGAGGAGAAACTAAAGCTNTCTGNGAA
AGTACAAAATGCACANGCTTANCTTACTCAGGACTCATACTCATCA

362
ATCCAACCTACTGCAGCTACATACAACGAGTTCCATTGTACATTGGAAGCACAGAAAGAAGTTGAAAAATTGGAGAAGTACTTGTCTTA
CTCAGGACTCATCGGGG

372
AAAACATTANGTGCATTCANGTAGAGGATACNANNANTCCACNCCATCNCNTATCACTCGACAGANANCAACTNCNAATACAAANCCAC
CAACTAGTATTCGATNATNCTNTTGGNTGCATGGAGGACATTNGCNATANCTTTCATCTCNCGAGGATGATGGGAAAANAAACTATAGC
ATTAGCAGTCNCNAGGAATGAAAGGAATG

373
AGTATCAACAACAATTGAGGTTTTATGCAAATCTCTTCCAGTGGAGTTTGCTTCATACTTGCATTATTGTCACTCTTTAAATAAGATCA
GTCTTACAGAAAATGAAACAAACAAGCATGAATGAGAGTTCCTCTCAGATGAAAGAAATCCTATTCAAAGACCCATTGGTCTGAGAAGA

AATTCTTTGAGAATTAACTCTAGAAAGAGGCAAGAATAACCTCAACTACTGATCATAAACCTTCTCAAAGGCTAGCTGATTGAGTAGGA
AGAACAGCATAATCCACCACAACAGCACCATCCAAAAACTCCTTACTCAGGACTCATCA

376
GGTTGGTGGTGGTGGCTATGCTCCTTGAATCTTGTTCTCTTCACAATTTGCAAACACTATATGATTTCCTATTCTTATCTACAATATTA
TTAATAGGGTTCTTGAAATCAACTGCAGGTCCCTCAGTAGAGCAGAGCATGAAACCAATCATGCCACTTGGATAAGTAGGAACAGTAGT
CCATGCATAGTTGACAGAGCCTTTGAAGATCTGAGGTCAGCATATTTTCTTTTTACTTAGGGGTGAATCATATATGGTCTGTACTCTGT
AGTATTTGCTCCTCGCACAAATCTGCTGCAATACAGAGTAGTATTCTCTATATTCCTGTAGGAGATGGATGGATGCTTACTCAGGACTC
ATCA

378
TAAGACCTACAAGGCTAAGCTAGTTATCTTCCCAAGGCGTGCTAAGAAGGTCAAGGCTGGTGATTCTAGTGCAGAGGAACTTGCTACTG
CCACCCAGGTCCAAGGTCCATACCTGCCTATTACTAGGGAGCAGCCTGCTGTTGACTTTGTGAAGGTGACCGATGAGATGAAATCATTC
AAGGCCTATGGCAAGCTCCGTATTGAGCGTACAAATGCTCGACATATGGGAGTCAGGTTGAAGAGGGCAGCTGAAGCAGAAAAGGAAGA
GAAGAAATAAGCAATTAAAATCCTGAGCAATCTCACGAACCAACCGCTGGAACGGAAGCTTCCTGATCAGAAGCTCAGTCGATTTCAGA
TACTTC

380
TATTAACCTCTGGCTATGACCAGNCTTACGCTGTAGATGCCCAATGAAGAAAAGCACAAGATTTCTCACCGAGGTAAAGCTCTTGAACT
AGTGAAGTTACACTTTGCTGAGGCTCGATACACTTTCCAGACCGACTCCACTACCTAAGACTACAATCTATGCCTCGGTAGTGACATGA
TCTTCATGTTCTTGCTCAAGAGCTATAATTGATGACAATGTAAGCTTCTTTTATTCTCCTATATAAAATATCGTGAAGGATGAGATACG
TGAAATTTTTAGAACTTATGATTATTGCTTATATAATTTAATTCAACTCCTAGAATATTTTGATATCTTATTCATGAAAAATATAATTT
GCTCATTTGCTAAGCTTACTCAGGACTCAT

381
GTTTGATAAATTCCTGCAAGGCTCTGTCAACCAACAACCAAAATATCGACCTCCTGGAAGGAACATCCCGAGCGATCCAATGGTTGGTT
CCAGAGTGCAGAATTTTGCCGTTTCTGATGCAGCTGACAAGCTGGGGAACATGAATATTGGCTCTGGCAGAGTACCTACAATGCAGCCA
GTAG

388
AGTCCATTATATGGATTCATGCACCTCGNGTTGATACCCATGCTAGTCGGATTTTTCACTTACAAGATTGCAACTTTTGTCCAAGCAAT
CGAGGAAGGTGTATCGATCATTGGAAATAAGTCACAAGCTTAGCTCTAGTTTTTATGATTTTCCATCAACCTCTAATGAAAATAGTAGA
GGAGAAAAAAGTTCAGGAAGACGTTGGATTCGAAAAATGAGCTACAACATCTATACTCAATTTATCACTGGATGGATTGAACAAATCGA
TGATTTCCTCATGTAAATTCTTATATATACTAGATCGTGCTCTCAGTTTCGTTGATAATCTCAAGTTTCTTATCCCGTTGAAGGTTTAC
TCAGGACTCATCA

389
AGCTTGTATGTAACCTGGTTGCATCTCTTACCCAGGGCCTTGTAGGTGCTGACCTTGCCAACATTGTCAATGAAGCTGCTTTGCTTGCT
GCTCGGAGAGGGGCTGATTGTGTGTCAAGGGAAGATATAATGGAAGCCATAGAAAGAGCAAAATC

402
GTTGACGTTTTTCCAGCTTTCTTTGGACATGGTTACTTTTCTTCTCCTCTGCAGCAGCCTCAGCAACCTCCTCTCCTTCCTTCTTGGCA
GCACTCTTCTTCTTGCGACCAATTTCAACACCATAGTGCTGGAGATACCACTGTTTA

408
GAGCTTTGATAATGGCCGTGACCTATATGTTTATACAGCATAACAATGTGTATCTCATGACTGCATCAAGGCAGAACTGTAATGCAGCT
AGTCTCCTTCTGTTTCTACACCGTGTCGTAGATGTATTCAAGCATTATTTTGAAGAGCTAGAAGAAGAATCCCTTCGCGATAATTTTGT
TGTGGTGTATGAGCTACTTTGATGAAATGATGGACCTTTGGTTATCCTCAATATACCGAAGCAAAAATTCTTAGTGAGTTCATAAAAAC
TGATGCATATAGGATGGAGGTTACACAAAGGCCTCCAATGGCAGTTACAAATGCTGNGTCCTGGCGTANTGAAGGGATAAATT

409
GTAGTGATAAAGGTGGAAACAAGTCCTAATTGCTCAAGCTAATGAGACCAGGTGGAAGGTTTCATCCTGATTTTCACATCACTCGTATC
GTACGTTTCTTTTATGTACATATATATATATATAATAGATGTCGGAACCCAGGATTACATACTTCTTCTATACATCGCATTGTTAATAGCT
ATGACAAGGTGTGCAGACTAGCAAATGGTGACAGTAAGCACTTGTTTATCTGAGAAATGATTTATAAAAGAGGACATATATCCTCTTAT
TTCTGCTGCATTGTTCCAGAGTTTCAATGTATTATAAGTAGTTTGAAGTACTCCTGAAAACTTTGGATTTTCAGCTTCTATTTAGGGTT
T

410
CTAATAATTTTGGTCGAGCTGCGCATTTTGCTTCTAAGAAAGAGCAATCAGCAATTCGTTCTGTTCTCAAGTGCATTGAAGAATACAAG
CTTGAAGCTGAATTTCCACCCGAGAACCTTAATACTAGGAGGAAGAATATCACATCCTCACTTCTTTCCGCTCTTTTTCAAGCCAGTAC
CGCCAATGGACCCTTTCTGAGCCTTTGCCTTGAGCTCCTTCAGGGCCTTTTCTTCTTCCTTCTTCTTTGCAAGAAAAGCCTTGTCATTC
TCATCATACTCCTTCTTTTCAGACTTTGGTGCCTTCAAAGGCTTNTGNTTTTCCACCTTGCTTGGAAGACATGGTTAGATTTTTAGGGC
TT

411

CATGCTAGTGCTTCTATTGCAGCATCCTTTTCTGCAAGGGCTTTGTTTCTCTTTGGTTTACCTACAAATTGCATTCCCTTGAACTCTGC
AAGTGCCCTAAATTCATTTGTTTTTAGATGCTTTGTTTTGTACTTGGGAGGGGATTGACCCGCCCTCATAAGTAGCGTCTGCAACAAAC
TCTTTGGGTTTGTCCCGTCCCTTGTAAATCTATCAGCGTCAGAATCTTTAGGCTTCTTGTTTTCACGACCAAACACAAACCTGCCCTCA
GACTGATCTCCTGAAACCAGTTCCTGAACAGCAAGCATAAGGTATTTGCCTTCCTTGTGGATGTCTACCTCAG

412
TCCACAATAGAACCATTGTTGTAGCCCTTATTATCCATAGCNTTCAATCTGGACCGCGGNAAAATAATACNACTNTTNAGNTCGTNCAC
ACGACCATCTGCTATNNCTGTCCCTNTCACCCCCAATCCCCAC

419
ACCACTTTATCTGGCAGCGGAGTCAGGTTTTCATGATGCTTTGATTGAAATCTTGAAAGTTTGCAAAGAACCAACACATGTTGCAGGTC
CATCCAATCGAACACCTCTACATGCAGCGTGTGATTCAAGAACACGCGGAATGCGCGATGGCACTATTGCATTGGAAGGGAGATTTATG
CGAAGAATATGATGTATGGGGTTGGAATTCACTACACTATGCTGTTACTCAGGACTCATCA

423
CTTCAACTGCCAAGGCTCGTTCACAAAATCAAACTGTTGTCGTCCAAAACCCAATGTCTGTCGATGAGAGTGGCAAGTTGGTGAGTAAT
GTAGTTGTTGGTGTAACAACAACATCATAGCTCGTCTGCTAAACTTGCTGTTCGTGGACAACAATTGAGAGTTTTGCTCGCAACAATTG
GAAA

431
CTTCTGTTGTACAGTATTCAGTGAACTCTACTGCACTTGTAGGAGACGCTGATTTAGATTTTGAAATAACTGAGAAGGGTTTCCAACAC
CCTTCTGAGGGACAATCAAACTTTGAACCAATATTTCGTTCTGGAAGTTGTGCTGAGAAAGGACCCAAACAGTACATGGAGGATGAACA
TATCTGTATAGATGATCTAATCATACAT

433
GATAAAGGTGGAAACAAGTCCTAATTGCTCAANCTAATGAGACCAGGTGGAAGGTTTCATCCTGATATNTCACATCACTCNAATNGTAC
GTTTNTTTTATGTACATATATATATATAATAGATGNGGGGAACCNAGGATAACANACTTCTTCTATACA

434
CTGACATATGGCTCCTCCGTTTCTTGCCTCGAGTTTGTGACCCCTCTCTTCTAAGAAATGTTTTTTATCATCCGAGAGTTCAATGTGAT
CGCCATCTATGCATGTCCAGTTCTCATACAGAACAACATTTGGGATTAG

436
ACCTTCTAGTGCTGAGGCTCCAGCTTGAACTCTAGTTGTTCTATCACTGTANTGTAAAATGTGCATCTGAATAAGGNGATCCCAAAAGA
TAANAATACTAATGTAAGGAAAANTGGTNTTNCNNGCCAATGCTAAATAANTCTACATAAACGTNACTCATAGACTCATCATCA

438
GCGGAGGCCCGTACTGTGTGNATAACGCTGAANAAAACTNGNANATACTGCTNGCNTNANCCNACNCTTGNGNATNATNNGTGTACCAA
GATGCCGTNTTGGGNACGACATTGACTGGTTTATGTNNNTACCTTNATGAAGATGCTNGCGTTNCTCAGGAACT

445
GATGATNTCATTCTCGTANTGCAATGCCAGANTTTTTGACCGCGTGACTGAATGGNCTGGCTTTNCTCAATGCGATNAATCCTCTCAAT
ACATGAGGCTCATGGGTTTCAAGTATTGGCTCANAGNACTCAATAGGTTACTCAGGACTCATCA

450
CGCTGNNGNAGTACTTGAGTNGTACAGTGTNGCCTTCCAANATNGNCATGGAAATGTNNAGTCTGNNGNCTACANAGACTGGGTCTTAC
CTGANCAAGCCCTTNCACCTGATGTT

501
ACTGTTTTCGAACCTCTTATATNTTCGCCAAAGACAGCGTAAGGCTCGTCAAAAGGTGCCACAAGCCTGATCGCAAAGAATTTACCAAG
GTTGCAACCCGTACGGCTGATCGGTNTTGTTGTGATGGGATTCAGTTGGATTTTTCGTCAAGTTGATATTCATTCCAATCAACAACATC
ATCGTTGGTGCTTCTTACTCACGACTCATCA

512
AAACGTTTTNCGTTGNAGTGAGGTTCACAAAGTGAACTATGCTAATGTATCCACTAACAACTACGCATTGGACGAAGTTGAAGAAGTGA
AATGAANGAGTGGACGTAATTAGTTCAAGGTTTTCCATATNTGTNATTGNGTCCAGCCCGTGTGCTTTCCATGTCTTTATCTNTAGTAT
ATATACATGGCTTTGAATGTGAATGTGTATCATAATTTTCTGGCAACTTTCTCCCCCCATTTCTTATTTAGCGTGTTGCCATTACTCGA
TCCCGTTNGTACGATCTTGCGAACATCATGTTGGAGAGGTTTGTGTCTGTATGACGGGTCTACACCTTACTCAGGACTCATCA

516
CATGATTTTGCAGCGGCTGTTGAGCTTGCCTAAATCCACTACTTTGCTGGTTTTGACCTTGAAAACCATAATATGTGTTAGCAGGAACA
GCTGACATTGTCCTTGAACCGGGTCCATGCCACATA

517
TGGCAGGNTATTGGGTGNTCATTCTTGAAACATACTCTCGTGTNCTGTTGAACTTGGCGTGCANNNTCCTGACTAGGATAGGACAGATT
CTGCACGANGACNTTCTGAGCANTCCCAATTCGCCTGCGGCATTCATGCCACCTTCCTGGCATAACAATTGCACGATTGACAGATNGTC

CCATATNTAGTCGGCGTGAGGCACTCACTTGATTGATCACATGAATAAAACAGATGGACGTTGTTGNGATNCTCNTCGNNCCAATGCTT
CTGATATAGATATNGAGTTTGCTGATTCAAGATNACTCAGGACTCATC

536
GAATTGAGANGCAATAATCCTTCTNATGTATATGGTAGCCTTGTTGTCCGAGGAGCACTGTACAATGACAAAANAATTGTATTTGTTTT
GTAAAACAGTACTCAAACTTGTTGTAAGGTGTCAGTATTTATTTTCAACCTATTTACGTANAACAGAATTGCCTGCTTGAATTTCAATC
GCCCTTTACTCAGGACTCATCAGACAC

540
CTCTGCAGCCCAAACTCTACTCCCTGATAGCGAAGGTGCTATTGATGGTCACCTTCGCGAAGTTGGGCTAACATTTCACTTGCTCAAAG
ATGTTCCTGGATTGATCTCGAAAAACATTGAAAAGAGTNCTACTCANGACTCATCATGGGGACGATGG

545
TTAGTTATATGTTGTGTTTGTTGTCTTTTTGTTTCTTTTTTCGTTTTGTCTTTTGGNGGTCTTGTATCGAAACTTCACTATCGATGGAT
TGTATTACTTTACTCAGGACTCATCA

551
TCATCACTGCAAGCCGTGCAACAGGAGTTTTGGTTCTGAAGGTGCTCTCGAATCTCACTCGAAAGCTAAACACAGTGCTGGAAAGTAAA
TAGACATGGAGACATTTAGTCTTTGTTTGGTTGTTACCTATGATCAGAAGAGAGCTTTTTTAGTCGTTTTTTTCCCCGTTTCGATTTGG
TTCATCTGATGTAAAGGAAAGATGAGTTACATCTATTATGATCTATCTCTAGGTCAATCATCTGTTTTGATG

555
TTGTAATACCTAATAATTATCTACGGAATCTCAAAACAAGACCAATAAAATACTGTCCTCTACAACTAAATTATTGATATCATAATCAA
ATCTACAAATAAATCAGCATATAAAAATGTGTGCGACACACCCACACCATGGATACACAATTCGCGACGAAATTTAGCCAAA

557
ACCCCATCAATTCATTGCCGCTAACAAAACAGAGTTGTGTAGTAAAAAAGTAAATGAAAATAGAACACTGAAACAAAGCTAAACACAGT
GATATATGTCTGAAAGCAATCCACTATAAATATGCTTAGCATAAATAAAAAAGAATCCCATTGCACCATATGTAGA

559
TTTGGTAATGACACATTATAAAGTACGATGAACTGACAAATGAGTTGTCTTTCCAAGAGTGGACTCAACAAATGAGGGATATGTTAGAG
GCAAGAAAGCGTGGTGACTTGGCATT

562
GGAGCAGTNCCTTTAGNGGAAANAGNTACTATTTNTTNTGGGGCNGACCNCCTCGGCCTGCAGACTNGGATGTATTACAAGTTTTTGGG
NGGACAATAGTNGCTGTNGAAAATTG

564
TCTTCTTTTACAGAGAGCCTGAAGAGGCAAAAGAGCAACAAGATGAGGAAGTACCTGCTATTGCTGATTATGCAGAATATGGTGGCGCT
GCTCTTGGTGGTGATTGGACTAGCAGCCAAATCCCTGATGCCCAATGGCTGCAGATGGTGCAGCATCTGTTCCTGCAGCTACTGGTGAC
TGGTCTGGAGATGCAGTGGTTGAGGGAGGTTGGGATGCTGCAGCTGCTCCTCCTGCTGGTGTACCAGAAGTGACCCCTGCTCCAGCTGC
TGCTACTGGCTGGGAATGAACTTGACTCCTTCA

568
CGTTGTCGATGACATGGTTTCTAGTAACGCCATCCTCTATGAGCCTGGAGAGCATCCTGACCATGTTGTTGTGATTACTCAGGACTCAT
CAG

569
AACAAAATCATCGAAAAAGTTGAGTTTGCTAGTTATGGTAATCCAGATGGTGCTTGTGGAAACTTGGCTGTTGGAACTTGCAATTCTGC
AAAGAGTATGGCTGTTGCACAAGAAAAATGCTTAGGCAAAAACTCATGCACCATCCCAATTGAGAGAGAAATTTACGACGAGCCGAGCA
AGGACCCATGCCCTAATATCGTTACTCAGGACTCATCA

571
TGTAGCTAGAAAGGTGAAAAAAACGGCCTAACATGCCTGTCTTTGCTAAGTTTCCCCCTCTTCACATGAAATCATAATCATCATAACCA
TCATATGCATTTACTACTGCATCATCATCTGCCTTATCAACATGTAGCTGCTTCTTCTTTGCACCTGTCTTCTTTTTACTCGCAGTGGC
TTCCTTTTCTGCTTTACTCAGGACTCATCA

573
ACCTTCTTCCAGTTCTTCTCTTTCTTTGGTACCTTTCTCATTCTTACCACCCAGGTCATACTGCTGCCCTTCATCATCAGAAGAGCTTG
CATGCTTATGTCTGCCTTCAGGTTCATTGTCGCTAGAATGTTTAGTCCTGTGTTTGTGTCTATCTT

576
TCAAGTGATGAAACCAACAAATATCTCATAGATGCTTGCGCGAAGTTTGAGATCAGATGTCCTCCACCTCTAACAACAACTAGATTACT
TGATAAACTTGTGGGACACTTTCTGGAGGAGACATGT

579

ACAGAATGGAAGTACACAACCAACACTCTATCCAAGATGGAATACACAAGAGAGAATAGTAGCCAATAAAGAAAAAGGAGGGGATAAAC
TTAGAAAAAAAATACATCCAAGAATAAATAAAACAACAGCTAGATAATACTCCATTAGGTGGAAATAAAGTGACTTAGTTATGGGCTTC
ATCACATATTCACCTTTTTTCTCACTTGGTCAAGACCCAAACAGCATAGACAATACCAGGTAGCCATCCAAAAAGAGCCAACAAAACAC
A

580
TGCTGTGAAGGACATTGGTACCAAGACCTAGTTGTGTGCATTCTGCAGCATAAATAATTGCTTTTTAGCGAAGACGTTTTATATCTTGT
TATCGTGGTACCTTTGCAATCTGTTTTATCGTGAAAACACCTTATATCTATTGGCATGGCTTGAATAGTTGAAACTCTAATAGTTTCTG
TTTGGCATAAGGCAATGAACTGGATTTGATAGCAGAAGTAATCTACATGTCA

583
ACTGCGTAGTGATCTGACTGCGTAGTGATCTGACTGCGTAGTGATCTGGATTCTTCCCTTTCTTATCTGT

587
ATGTAGAACTAGCCTCGGTTGTTATTATCAAGTCTAATAATCTGTTTGAAGGGACAGAATGTGGACTACATTTTTTTTTCTACTGATAT
TGCGTTCTTCTGTTTA

592
TGGTGGAATCCAGGGCGGTATATCAAATGGAGAAGTTATCAATATGAGAATAGGTTTCAAGCCAACTTCAACTATTTCTAGGAAGCAGC
AAACTGTGACAAGAGACAAACACGAAACAGAACTCATCGCTAGGGGTCGCCATGATCCTTGTGTGGTTCCCCGAGCTGTTCCAATGGTT
GAAGCAATGGTCGCCT

595
ATGACTGAAATNCACTNGGTAAGTTTNTTATGGTNGTACTGCCNGTGACTGCTNGAATAAGGTNNTNTCTNACTTTCNACGGNTGGAAG
AGATACAGTGGGTGCATGCTNNNTGCCCGGAATGAGGAATACACAGCCGAATCCACCANTGTTACTCANGACTCATA

597
CTAAAATCTATATGCTGCAAAAAAGCATAGAGAATCCCAAAATTATGTCTAGTATACATTTCCTATACTAGACTCGGTAACTACCAAGG
CCTTCTTGAACCGCCAATAGGAATTACATTAGACC

598
GTAATTTTGGAAGGGATGGGTCGGTCTTTGCATACCAATTATAATGCAAAGTTCAAATGTGATGCTCTCAAGCTTGCTATGGTGAAGAA
TCAGCGATTGGCAGCAGAATTGGAA

600
CCACTCTTTTGCTGGTATTTATATGGTATGGGATATTTAGTGAATTGGGTTTTTCCTGTACTGCTTTCATCTTTCATTATGGAGTAATA
ATG

609
TATCTTTATTGNAAGTNGCTATTNNCAGTGTGCTGGTCAATTAGCNAGANANNGATGGCTGCAACAGGTCTANACNTGCAGCTNCTANT
ATTTCATNGCCTGGATCAGAAGGTTTGTATTACTCANGACTCATCATCATGAG

611
TGGTTCTCCTTATGCTTATGGTGTTTTGGATAATGGGTACCGATATGATCTATCTGTGGAAGAAGCAGCCGAGTTGGCTAGACGAGCAA
TTTATCATGCTACATTCCGTGATGGAGCTAGTGGAGGTGTTGCTAGTGTTTATCATGTCGGACCAAATGGATGGAAGAAGCTATCAGGT
GACGATGTTGGAGAACTCCACTACCACTACTATCCCGTCGAAGTGGCAGCTGTGGAGCAGGAAATGGCTGAAGTTCCAGTTGCCTGAGG

628
TTTTCTGTTGCTATGCAGATTGTGTTTTGTATTGATTTGCGGTGTTTTCTGTTGTACAGGTGTTGGAGTTGGCTGGAAATGCGGCAAGA
GACAACAAGAAGAGCAGAATCATTCCTAGGCATGTGCTTTTGGCAGTGAGGAATGATGAGGAGTTGGGAAAATTGTTGGCTGGTGTTAC
AATTGCAAGTGGAGGTGTTCTTCCTAACATTACTCAGGACTCATCAT

639
AATGACAAAGATAGTTACACTCGCAATACTTGCTTATGGACAGTCAAAGCTTGCTAATATCTTACACGCTAACGAACTTGCAAGGCATT
TCAAGGAAGAAGGAGCGGAGATAACTGCTAATTCACTTCACCCGGGATCAATTACTACCAATCTTCTCCGTCACCAAAGTTTGGTTGAA
GGCTTAGTTAACTGGATTGGCAAATACTTCATTACTCAGGACTCATCA

645
CCCAGCTAGTGGTCGGTCATACCATACAAAATTTGCACCTCCTAAAGTACCTGGGATAGATGATGTCACTGGAGAGCCATTGATACAAC
GAAAAGATGATACTGCTGCTGTTCTCAAGTCAAGGCTGGAAGCCTTCCACAGGCAAACTGAGCCGGTCATTGACTACTATGCCAAGAAG
GGTAATGTAGTGAATCTTCCTGCTGAGAAACCACTACAGGCAGTCACAGCTGAAGTCAAGAAAGTTCTCTCCTAATGGAAGGATGCCAA
C

648
GTACTTCTCTTTGATGCATCATATCGTCTTGGCCATGCTAAATTTGATCTTCTNGTTGGGTTCTTCATAGTTAGAAAGGTAAGACTAGT
TATATCTGTTGCAATATATATTTGCTACGTGCTAGTGCATCTCAAATGGTTGTGAAACTTGTTTGCACACACTGCTGGAGTTATATGAT

GGTCAAAGTCGTCAAATCCACATTCCTTCAAACTGGTTCTTTTTTGCTGTTTTCTATGAAAGGTTCAATTGTTTACTCAGGACTCATCA
T

664
CCACCAGACCAGCAGAGGTTGATTTTCGCTGGTAAGCAGCTTGAGGATGGCCGCACCCTTGCTGACTACAATATCCAGAAGGAGTCCAC
CCTGCACCTTGTCCTCCGTCTCCGTGGTGGTT

667
ATTAGAACTATCATGTTCTTGGGGTTTTGGACCCATTCTTAAATAGAATTTTTTATTCTTACTTATTCTGAGTCTTTGCTAAATACTTC
AACTATTCAAATCACGAAGTTACGATTGGTCAAATGATATAAAAGGGA

673
CCATGCTGGCANTCTTTGCTGTAGACATATTTNGNTTATTGTNCTCTGTCATTTTGTTCAGTGGACTTACTCACGCACCTCATCANGNN
GNNGACAGAACACAATAACCAAAATATGTCACAGCAAAGATGCCAGATGATACTCCATTACAAGAGGGGATCCCTACGC

677
ATGATGGAGAGAGCTACCAGCACTGATCTGTTTCCCAGTTCCGGGTAGTATAGCCGGTGAATGCCGACATTGCTGAAGGAGAACAGCCG
CCGGAGTATAAATTGACGTCCCGGCGCCTCCGGAATAGGCGGCGGTCATAATCGGCGCGTTTATCCG

683
AAATCTTGGATATTGAAAAGCTCTCTCTTTTGCATTTCCCTTGTTCAAGAATCTTGTTGCTTTTCATTGGTTTTTCAGCAAATTTGAAT
CCCTCAAATCCATTGAACTCCCTTGTTGAATCACATCCACCACATTCAGACATTGCTTGTCTATGATTTAGCTTCTTGATTGCTTGCTT
CTTTGACGGTGATTTCCTTACCGGAACATGTAAAGCTGTGGTCTCATCTAATACATAAGCAAATGATCCCATCGAAAAACACCTTCTTG
AATCAATATCATTGCTACTCCCTTCATGTTCATCAACACTACCTACATGATGTTGTTCTTG

684
CTTGGTACAGTNTTTTNAGGCCACTGACCTGATTCNTAGCNCTATACTTACTACCTTGTCCTGGCTGTANCATATCGCAATTGCTCATN
TATANCATNATGTGGAATGGAGATAATGTGCTGGACTCCTNATTCAAGTTTNCAAANCTAGAGGGGGCACAAAGGGAANGNGNCATGTC
TTTCCNNGGCTATAGCAAGNAATGGTGGGATNAGNNCATTAAACACATGNCTGGCANNTTGCAGAAGGCTGTTTGATCAATAGTTCAAT
NATTGANCACAACTAGNCCATCATTTGTATAAANTGCTGAGCCATGCTTGCTCTTGATTTGATGTNACTCAGNCACTCATCA

685
CGTGCTTGGATTTCCAACTGGAGGGCCTCGTATATTANTACTTACGCCTTCAACCCAACAACAATTATCATAATCATCTTCACAGTGGA
GGTGGCTCTGATATTACTACTTTTGCTCTAGGTTGAGTAGTAATTTTCACCTTATTGTAATATTGTCATACTTTCTAAATAATCACATG
CAATGTTTGTTCACTAGANATGCATGAGAGTACTGACTCTTTAGACTATTCTCCTACTTTTGCTTAATCCAGTGTGTTACTCAGGACTC
ATCA

686
CAATAACAGAGTAGTAGATTCATAATATTATCGTTCACATGACCACACAGGCTGCAGGATTCTCGTCGCTAAAGGCAGTAGTATAGCGA
ACTTCAGTAGAACTGGCACGAGCAAGTTGGTTCGTTTGGAAATCATCTCTTCGCACGTATCCAGCAGGAGCCTTCTTATCATAGACACC
CTGTGCATAAGGATGAGCAACCACATCGTATGGGACATAGGGCCAAATCT

689
AGTAGGCNTTCGCNCAAACTACCTANGGTGNTNGTTGGTNGNCAATCNGCTTGNGGACGNCNNGACCCTNGCTGATTACAANATGCAGA
AGGAGTCNCCC

801
TCTGAAGCCAGGAGCGTTAGCCCAAATCAGGAATTCCAAANTCACTGCTAAATCAAAAGTACTGAACAATCCTAAAACTCTAATTGTGA
TGAACCACCAAACATCCGATTCAGGAATCGGAGTTTCACAGCCAGCACCAATCATGGAGAGCGTTCCTTGCTTTACTCAGNACTACATC
A

805
GGGACAACTGGACATCAAGGAAAATTCTCCAAAGATCAGGCAAGGACTATACCGTTCTTTTGGTCCTTCCATCGGGTATATATCATTAC
AAATTCATTGTGGGATGGGGAAGTTAGATATATTCCAGAACTTCCATGTGTAGCAGATGAGACAGGCGTTGT

806
GATGGTAACACAAAGTATTTTGCACCTGCTTTTACTGGATTTCCATCACTGTCAACTACTGAAGCATATGTTTTACCATTAGGAACTAC
TTCATCAGAAGGAAGGAGAA

811
CCCTTGGTTACCTGTTTGGCGATGGCAAGAACTAGTTCCTTCTGTCCAAAGATGAAACTGTGTAGTCATGAAAAAACAATGAGACTGCT
GTTACTCTATGTTGGTTTATTTTGTGTCTGGAGAACCAGTCTCGGCATTCAGATGATCCAATTGTAATATGATCATCCTTTCCTATTCA
CTGTTGGGATATATACCCAAAGCTTCAACTGTGAACTATCCTCAATGCCTG

813

GCCTTGAGGAGCCATGAAATCATTCAAGTCAAAAGGCGGTGATGTTTCATGGCACATCCATGATGAGAGATCGCTTTTAGCCCTTCAGG
GACCCCTTGCTGCTCCAGTTCTTCAATATCTGACAAAAGATGATTTGAGCAAGATGTACTTTGGGGAATTCAGGGTTTTGGATATCAAT
GGGGCACCATGCTTCCTCACAAGGACAGGGTATACCTGGTGAAGATGGATTTGAAATTTCAGTACCTTCAGAAAATGCTCTTGATCGTT
GCAAAAGCTCTTCTTGAGAAATCAGAAGGGAAGATTCGGTTCTAGGACCTCCAT

814
GCTGCCCTCGAGAAGGATTATGAGGAGGTTGGTGCTGAAGGTGATGATGAAGCAGATGGTGAAGATGATGAGGAGTACTAAGCCATGAT
GTAGGCTGTAAAATGCCGGTCTGTTTTTATTTTCTTCAGTGTCTTTATGTTGCACTAGTAAAAACTTTTATGTGGGTTGAATTCTGTTG
GTGTTC

815
ATGACAGGCGTTCTGGTAAGGAACTTGAGNAAGGAGCCCAAGTTNTTGAAGAATGGTGATGCCGGTATGGTTCNAGGGGACTCATCA

816
CTCAAAANAGGGTTTGNTGCATGTTCACTTCTATGGGGAGCTTCACAGGATCATATGCATTTGCTCATGGAGATCACTTTCATGAGAAG
TAACACAATTTATTTGTACGTACAGTTTACTCGGGACTCATCA

823
TCCAGTCTGAAATAAACGAAGACGTTATCACTACCTCTTTTTACAATACAAAGCATAAATGATAAACGCTACATAAAGCATAGAGTAAA
ATAAGAGGATCATTTTTCATCCTTGAGTCGAA

824
ATTCTTACGTAGCACCATCCTGGTGCTNCGCCAANNACCANTGCTGCATGGTGAATAGCTGAGGAAAACANTTGGTGCACCTGCTTACA
TGTGAATGTANTTCAAAACCACAGCNNAACNTGAAAGCNGTCTTTGATGCCACTNTCANGGTCANTGCTCCAGCCACCCAAGCAGAAGA
ANAAGAAGGGGAAGTCCCAAGNGGCATGTTCTATNCTGGGATCAACAGTAGGGTCCAATGTNATTACTGGTCACACATNNNNCCTTGTN
ACCTCTCTCTCNTATTCCTTTGTCATTTGTCTCTGGAGGAAGTAGGAGGTCCATGTGANTATGTNATCATTTACTCTGAACTACATCAA
T

825
AGTCCTTAGGCAATCATCTNCAGAAGCNTGGTCATAGCNGCACNGCGATCTCGCTATCGGATNCGTCGGNTTCGGGGCAGGTNTCGCGA
TTTGTNNGCNATAAAGGTGTATGTTGCCACACCTANGGATATTAGCTCTTTCNTGTGGTNTAAGAANGACCCGGTCTGGGTCNTAGGAA
TTATGATNATACTTGTAGGAAACCCATNTGGATTTNGTTGTTACCTCCCACGGGACTTCC

826
GGGAATGGAAGCGGACACTAGGTGGCTTCTCTGGATAATCTTTATCACAGAATAACTTCAACTGATAAATGCGACCTTCATGAACGGAA
TTGTGAGGACCAATAATGGTGCCAGTCCAGGAACGCATATAGATATCATCTCCATCATCCATCCCGTAGCTTACAGTCCCATCTCCAAT
ACCCTTTTCACCACGTTCAAGTTCCTCAAGTAATCTGAAATTCCGAGGGACCACAACACTA

827
TACCAGAAATTCAATTGGGTGTTACTTACAGGCATCCTGATGGCTCAGCATTACATTCTTTTCCCTCTGATCTTCGTCTTCTTGAGCAA
ATAAAGGTGGAGTATGAAGTTTTGCCTGGGTGGAAGAGTGATATTTCTTCCATCAGGAAGTATTCTGACTTGCCTAAGGCTGCACGCGA
GTATGTAGAAAGGATAGAGGAGCTTGTTGGGGTACCTATCCATTACATTGGTATAGGACCTGGTCGTGATGCCCTTATCTACAAGTGAT
TTTAGATTTTTATCATCATATTGCCAGAGTCA

832
GTATGTTCCTCATTTGAGTACTTTATGTTCTGAACATCTCAATTAGACATTAGTATAAAACTGTGTTTTTCAGACAAGTATTTACTGAT
GCATTTTGTTGCCATTACTTCTTTATACTTTACTGAAAGGTGTCGATGCTGGTTTGAAATCTGACATTATCTGTAGTTCTAAAGTGATA
GTTTGCAAATCACTGAAAGGAGGAAATTTTACCTTACTCAGNACCTCATCA

835
AGGTTCGATCAGCATACTGTAAGGACGACTTTGAGTGGGATAACTTGAAGCGCCTAGCATCAAAGATGGTGGATGATTCTAATAAAAAG
CTAATGACGGATTATATCGTGGATACTAGCTGCATTGCAGATGGAAAGCAGATATGCAACTAATCAAACTCCTTACTCAGGNACTCATC
A

836
GCAATTTTCAAGTGAAACTAGGCATGAAATGTTTTCCTCTGCCTATACATACTTTGGCTATTTTACTATACTCACAGAATATTCACGTA
TAGCAAGTCATTTGCTAAGTTAGAATCTCGACACACTGGAAGAGGCGGCACCAACTGATATACATACCATCTGCATTCA

839
CATCAAGACCCTTGCATATATGTTTTTTGAAGCTGGAGTTTAGATTTGGATCGAGCAACTGATCAACTCCTTTCTCGTTCAACGTTGTG
TGTACCCAAGTAGTTA

840
CACAGGAAGGACTAATGGGGTTCATGGTCAAGCATTCTACTAACTNTGGTATTTGTTGAAACAAAGAAGGGTGCTGATGCATTGGAGCA
TTGGTTGTGTATTACTCAGGACTCATCA

843
CTATATTTGGTGCAAGGGAATATCCAACGAAGTAGTCATCGAACTAGGTTGATGAACCCAAGTTTAGTGAAAAGAGCTTGAACCAAACA

846
TTTTTTGTAATGTGAGGATAAAAATTATTATCTCTTTACAATTTACATAATAGACTTATTTTGCTTACTCAGGACTCATCATAA

847
TAGTAGTNTTATATCAAGGAAAGGCTTCTATTTTACATGGATGATCACTCATCTAGTATGAACTTATATCAGTCGATCAATAGTTTATG
GGCGATAGATTTTTCCCTTTTGCTTACTCAGGACTCATCAATCA

848
ACCATATATTGTGCTCAGGACTTCTGTACGCATATCTGTTGTACTTGTCTTGGACACCAGATACAATTCGGCTGATGTTTGCTAGTAAA
TACTGGCTTCCAGAGGTTTGTTCTTGTACTTAACAAATGATAGTTGTCATCTTGTGCTGATACCGTGATTTAGGCTGTTGACATTTCAT
TCTGTTCAGCTGTCTGGTATAGCAAAGATGTTCTCCAGTGAGATGACATTAGCATCTGCTTGGATTCACCTATTGGCTGTA

850
TACAGATGTAGTGGCTGGTGAACCGCTTCTCCTTCTTCCACGAAGATTCAGACAAAACCGAGCTTGGATGGAACTGAACAAGATTGAGC
GA

851
TAATGTAATCAACATCATTTCCTTGACCCAGTTGTCCGCCAGAAGGATGTCCATCTACAGAAAACGTCCCATGGGAGCCTCCCCATCCC
CAAGTGTAAACATTTCCTCCAGAAATTGCTGCTGTATGCTTCCAACCACAGGATACCTCAGAAACTGAATGCCTCAAAAATGGTCCTTC
AACTCTTTCAGGTAAATGAGCGACATCTG

855
AGTATCATGAGAAGTGCTGAGAGTTTTTGGGTGGAAAAAAGCAGTAGAAAATCTTCATGTAAATAGTGTTTTTTTTCCTATGCGAGTTG
GGGACTTGCTATCTAT

856
ATATATCATTGATGAAACTGTTCATAATCCCCATAGATTTGCTTGAGATACCAATATCTGGGTGAACTTGTTTCAGAACCTTGAAGATA
TA

858
CATAGCTTTGATATCATCATCATCAACTTGAAGCCCAGCCCAACTCAAATAACTTTTCAAATCCTCAACCCCAACTTTACCATCTCCAT
CTTTGTCAATCACCTTGAAGACATCCTCCATTACATTCATTTCTCCTCGTAATGCTCCGTTTTTCCTCTTCTCCTCGCTGTTTCTGCTA
ATAGAACTATCCAAAACCTTCTCGAAATCCTCAAATTCAACGTATCCATCTTCGTTCGAATCCGCCACCGTCATCATCGTACCAATCAT
ATCGTCGCCGACGAATCCACCGTACGATGTTCG

859
ATCTGACGAAGTAGAACCATCTCTATCAAGATAACAGACCCATTCTCTGTACTATCAATAGGATCAATTATAACAAGTCACACACTCAT
ACTCCGGAAATACAGAAACAAAGAAATTCCACGATCGAACTACCAAAAGAAAATAGAGCGGACTATAAATCGGAGCTCTAAATGATTCA
TTTTGGACTCAAAAGAACGGACTTTGCGGTCCTTA

863
ATTTCCCAAGTAATCAATTCCTCTTGNGGATACTCAGGACTCATCAT

864b
CCATTTACGTAACGTTTGAGCTCAGAACTAACACCAAAACCAACACCAACTCCACTTGCTAGGAAAAAACTTAGCACCTTATCTACGAA
AAAATCCAAGCGGCCAAGGAAGTTGCCATGAAAAACTCTTTTCCCTGTCACGGCGTAATACAATGCAAATGGCAGTTGAATCAATGAGT
AGATA

864
TAAATCAGGTGCTTCAATGAAGTGTTCTGGCTGTCAAGGGTCTGGAATGAAAGTCACTATTAGACAACTTGGCCCATCCATGATCCAGC
AGATGCAGCACCCTTGCAACGAGTGTAAGGGTACTGGTGAGATGATCAATGATAAAGATAGGTGTGGGCAGTGTAAAGGTGAGAAGGTT
GTGCAGGAGAAGAAGGTGTTGGAAGTTGTTGTCGAGAAGGGTATGCAGAATGGACAGAAGATCACATTCCCGGGCGAGGCTGATGAAGC
ACCTGATACCGTCACTGGGGACATAGTTTTTGTCTTGCAACAGAAGGAACATCCCAAG

867
GATGCTGATTTGATGAAATATCATACAGAAGCAATGCAAACGAACTGATGGGTCTGGTAGTAATGTTTGCCTCTACATCTAGTGCTTAG
TGGAGCCACACCACACTACACCACACCACACACAGACTCTCTGTACTTTGGCCTTGTCTTTTCAAGTTGCAAGAAAGAAGTACTTGTTG
GAAAATTTCAGCCGTTCCTGTATGGGTATTATGC

868
TTTTGGTCTGATTCTTATAGTTGTTTCTGGNTGTCTTGCGCAGTATTTCTCATTTCTAGTTTTCTCAGGCAATACTTTGGTCTGATAGA
GTTGCCACATACAATTATCTTTCATGTTCCAGATTTTGGTTACTTTGTGCCTATATACAAGGATCAGCTGTTTATGTTTTTCTTGATA
TTGATTAGTTTCTTTTTGTTTACTCAGGAC

872
TTCTTCAAGTTCTGAGCAGGTTCATCCTGATATCGGTATTTCAAGCAAAGCTATGGGTATCATGAACAGTTTCATCAATGATATCTTTG
AGAAACTTGCTCAGGAAGCTTCCCGACTTGCCCGCTACAACAAGAAGCCTACTATAACTTCTCGGGAAATTCAGACGGCTGTTCGATTG
GTCCTTCCTGGTGAATTGGCTAAGCATGCCGTCTCTGAAGGCACAAAGGCTGTTACCAAATTCACTAGCTCTTAAGAATTTGAATATCT
TTACTGTGTTAGGGTTTGATGATAGGTGTAAAAACAGTTCCTTGTAGTTGTACGCATCTTGTGTTATGTGGATTGCTAATCAATCATCT
ATGAAATTTGGGTTGTTACTCAGACCTCATCA

873
TATGGGTAAAGAAGTTCTGGGTGAGGAATCTTTCATGGATTTGGCTTATCTTCAGATGAATTTTCCTGGAATGTCAGATGAGTCACTTT
CTGAGGTCTATTTAGCAAACAGTTGTGACATTGATGCTGCGGTTGACATGCTGAATCAGCTTGAGCTCTAC

875
TGGCACAGTGACAAGTGGTTCCCTAGCTATCGCTGCATCTACGTTGGATTCGTTACTTGATCTCATGGCTGGTGGTATACTCTGGCTCA
GTCATCTGTCAATGAAAAATATCAACATCTACAAATATCCTATAGGGAAGTTGCGAGTTCAGCCAGTAGGGATCATTATCTTTGCTGCT
GTTATGGCTACACNTTGGCTTTCAGGTGTTTATCCAGGCTGTAGAACAACTCGTTACTCAGGACTCATCA

877
TTTTGCATCGACATTCATCTTGNTGTGCATCATTCTTGGAATTGCAGCATATTTGTACAATGTCTTGAAGAAGTAAAACTCCGCTAAAG
TACCTGTTGGATCGACTTCTATGTTGGCCTTCATGATTCATTTCTGTTTTTTTTGGGTGTGTGTATGCACGGTATAACAAGTTATTATC
AGGNGGTTACTCAGGAACTCATCA

879
TCCGGAAAGTGGCGGTTGATTGAATGAAGCCGAACAAGCGTCAAAGGTAATACCTTATTCTTTTTGTCCCTAGATTCGGGAATGTGATC
TTCATTGTCTTGAACATGTTGAGTTTCTTTGTCAATCTGGTCTGTGGTCGGGGTGGCATCGAATTCTGGAA

891
GCATGAANATGATCCAGACTCAATCTTGAGAAGCTATCTCTAACTGTATNTCAAGAACTGTGTGTNAGTATGTCTGTTGTACCATAGTC
TGATTCAATAAAATCGNATATAACAACGTATGTATGCAGCTGATTACTCACGGACTCATCA

904
GCTGGCCAGAGATCATACGAACATTGAGATGGATGCTGAACGTTTGAACCTCATATATATAAATCTTCCAACTGTGCAATGATGCTTGC
AAAGACCTGATAACCTTGAGACATGTCAACCAGTAGACAATATTCATTGTCTGCATGGTATGTATCCATAATTCCGTAACCAGTAGCTT
GAACATCATAACCCCTATCCTG

916
TTAAGCCTCCAAAGATTCTTGTCATTGAAGGATTACACCCCATGTACGATGAGCGTGTGAGAGACCTCTTGGACTTCAGTATCTACTTG
GATATCAGCAATGAGGTCAAGTTTGCTTGG

917
CCATTCTCAACTGACTATGCTGGTAGATATGTATCTGCTGGAAGTATTGAATACTGGCCGGACCCACAACGTGGAATCCGAGAGGCATA
CAGAGTTCTGAAACCTGGAGGAAAGGCGTGCTTAA

920
GGGCATGCACATTCTCCCAGAAAGTAAACCATCATTCATCAGCCTTGGTACACAAGTTCGAAATGGAAAAAACATAACTACATCATAGG
TATAGCAGTAAATGGTCTCTCTCATGCTAAACAAAAACTCACTTTTTCATATTCAAATGAGATGCGGGTCTTAAACTTCAATTAGCACA
CCAATGCGAGAGTCATGAATGTATGAACCAATTCTCCCCTCAGCAGCAAGTCGGCTAGACTTTTTATCGGCTGTTGAAAGACCCTTCTT
TCGGAGGTATTCCTGTGCCTTCTC
